# AI dialogues in cartilage repair: which guides evidence-based decisions better?

**DOI:** 10.3389/fcell.2026.1768270

**Published:** 2026-05-29

**Authors:** Senyang Xiao, Hui Li, Dan Xing, Jiao Jiao Li

**Affiliations:** 1 Peking University People’s Hospital, Peking University, Beijing, China; 2 School of Biomedical Engineering, Faculty of Engineering & IT, University of Technology Sydney, Sydney, NSW, Australia

**Keywords:** cartilage surgery, cartilage tissue engineering, ChatGPT, deepseek, machine learning

## Abstract

**Purpose:**

This study compares ChatGPT, DeepSeek, and Google Search in addressing cartilage repair-related questions across two domains-cartilage tissue engineering (CTE) and cartilage repair surgery (CRS)-using a dual-axis framework that integrates classification, blinded quality scoring, and readability analysis.

**Methods:**

Google Search was queried for the top 20 frequently asked questions (FAQs) in each domain (CTE in 2023, CRS in 2024). The identical Top-10 Google-derived FAQs per domain were subsequently submitted to all three platforms-Google, ChatGPT (GPT-4 API), and DeepSeek (V3 API)-enabling a matched three-way comparison. Questions and answer sources were classified using a modified Rothwell taxonomy. Answer quality was independently evaluated by three blinded raters using the Accuracy-Safety-Hallucination (ASH) framework. Readability was assessed via the Flesch-Kincaid formula.

**Results:**

In the CTE domain, DeepSeek achieved the highest Accuracy (median 5.00, IQR 4.67–5.00) and significantly outperformed Google (median 4.00, Bonferroni-corrected p = 0.036), while ChatGPT (median 3.67) did not differ significantly from either platform. In the CRS domain, both ChatGPT (median 5.00) and DeepSeek (median 5.00) significantly outperformed Google (median 4.17; p = 0.024 and p = 0.045, respectively), with Safety significantly favoring both LLMs (Cochran’s Q p = 0.018). ChatGPT and DeepSeek did not differ significantly in Accuracy in either domain. Readability analysis paradoxically favored Google (Grade Level 12.6–13.2 vs. 15.7–17.4 for LLMs), attributable to extreme snippet brevity inflating formulaic scores rather than genuine comprehensibility.

**Conclusion:**

ChatGPT and DeepSeek outperform Google Search in accuracy and safety, yet their value lies in complementary functional roles rather than direct competition. ChatGPT’s policy- and education-oriented framing and strong CRS safety profile position it as a practical tool for patient education. DeepSeek’s technical depth and academically concentrated sourcing make it better suited for clinical decision support and research. Google offers the highest readability and closely mirrors patient concerns but carries measurable safety risks in surgical contexts. These findings advocate for stakeholder-specific AI tool matching rather than one-size-fits-all recommendations.

## Introduction

Rapid advances in artificial intelligence (AI) have revolutionized medicine and medical education (Dubin et al., 2023). Notably, large language models (LLMs) such as ChatGPT (based on the GPT-4 architecture) and the open-source model DeepSeek have been increasingly applied to extract information from literature to aid medical decision-making (Praveen and Vajrobol, 2023; Xiong et al., 2025). Although DeepSeek shows promise in specialized contexts, studies note limitations in answering clinical questions for conditions like osteoarthritis (Wu et al., 2025).

The impact of LLMs is particularly notable in clinical practice, informing decision support, trial recruitment, and health research (Groot et al., 2022; Biswas, 2023; Sallam, 2023; Patel and Lam, 2023; Xue et al., 2023). Importantly, recent literature highlights the direct clinical importance of AI in “patient-facing” applications. These include directly answering clinical queries from both adult and pediatric patients (Pereira et al., 2025; Pereira et al., 2026), improving the readability of medical education materials (Reaver et al., 2025), and ensuring accuracy in orthopaedic medical coding (Fulkerson et al., 2026). Ultimately, these AI methods mimic traditional patient-provider interactions but leverage different prompts to bridge the gap between complex medical knowledge and public understanding.

Simultaneously, AI is increasingly converging with tissue engineering (Guo et al., 2022), which is critical for musculoskeletal repair (Kang et al., 2016). Due to its avascular nature, damaged articular cartilage lacks self-repair capability and is notoriously difficult to treat clinically (Bentley et al., 2000; Chen R. Y. et al., 2023). While traditional cartilage repair surgeries and emerging tissue engineering strategies strive to restore native function (Li et al., 2023; Zhao et al., 2024; Kim et al., 2019), patients and caregivers are increasingly relying on online search tools and LLMs for information on these evolving treatments.

We hypothesize that LLMs, such as ChatGPT and the novel DeepSeek model, provide more comprehensive and clinically nuanced responses compared to traditional web search tools like Google in the domains of cartilage tissue engineering and surgery. However, overreliance on LLM-generated content without professional verification could introduce risks. This study aims to fill a critical gap by evaluating and comparing the utility of these platforms in delivering evidence-based, clinically relevant information for patient education and surgical decision-making.

## Methods

### Study design and data collection

This study compared three platforms Google Search, ChatGPT, and DeepSeek on cartilage-related questions across two domains: cartilage tissue engineering (CTE) and cartilage repair surgery (CRS). Initially, Google FAQs were collected independently for each domain (CTE in 2023, CRS in 2024) to establish reference question sets. For each domain, the top 20 FAQs and their Google-sourced answers were recorded, along with source URLs. In the original within-domain comparisons, ChatGPT (GPT-4) answered CTE questions, and DeepSeek (V3) answered CRS questions.

To enable direct head-to-head LLM comparisons and address the asymmetric design limitation, a cross-validation was subsequently performed. The identical 10 Google-derived FAQs from each domain (the Top 10) were submitted to all three platforms: Google, ChatGPT (GPT-4 API), and DeepSeek (V3 API). Answers from the original within-domain comparisons are provided in Supplementary Table S3 (ChatGPT for CTE Top-10) and S6 (DeepSeek for CRS Top-10). This generated a matched three-way comparison on identical questions for both domains. Throughout, LLMs were prompted to simulate a Google search (e.g., “simulate a Google search for ‘cartilage tissue engineering’“) because they lacked live browsing capability; thus, LLM-generated FAQs represent model-internal approximations, not real-time web retrieval. All answers were saved verbatim.

**FIGURE 1:**
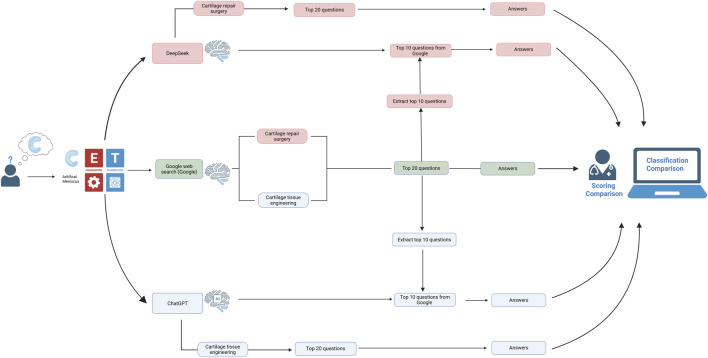
Study workflow. Google Search was queried for “cartilage tissue engineering” (CTE) and “cartilage repair surgery” (CRS), yielding 20 FAQs and answers per domain. ChatGPT and DeepSeek each generated 20 simulated FAQs and answers for CTE and CRS, respectively. For cross-platform comparison, the Google-derived Top-10 FAQs from both domains were submitted to all three platforms. All outputs underwent dual-axis analysis: Rothwell classification of questions and sources, and blinded ASH scoring (Accuracy, Safety, Hallucination) with Flesch-Kincaid readability assessment.

The complete study workflow is illustrated in Figure 1.

### Inclusion and exclusion criteria

The inclusion criteria for this study were questions directly related to the term “cartilage tissue engineering” and “cartilage repair surgery”, while the exclusion criteria were repetitive questions and irrelevant or non-specific questions (e.g., “What can cartilage tissue do?“). This study was deemed exempt from approval by the Institutional Review Committee.

### Classification of questions and answer sources

Two independent reviewers classified all questions using a modified Rothwell taxonomy (Fact, Policy, Value and 11 subcategories; Table 1). Answer sources were categorized into six mutually exclusive types: commercial, academic, medical practice, government, social media, and single doctor practice. Because categories can overlap (e.g., an academic medical centre may function as both an academic institution and a clinical provider), each website was assigned to a single category based on its primary institutional identity as stated on its “About Us” page. Institutions identifying as universities or degree-granting bodies were classified as academic (including academic medical centres); government-affiliated health authorities as government; websites whose primary stated purpose was clinical care delivery without formal academic or governmental affiliation as medical practice; and individual surgeon sites as single doctor practice, regardless of adjunct academic appointments. Ambiguous cases were resolved by consensus with a third reviewer.

**TABLE 1:** Classification rules of questions and websites.

Question topics categorized by Rothwell’s classification	
Fact	Asks whether something is true and to what extent, objective information
Education	Educational, cannot be classified into other subcategories
Accessibility	Where and how can patients get the treatment
Timeline of recovery	Length of time for recovery milestones
Restriction	Restrictions to activity or lifestyle during recovery
Technical details	Details of the treatment procedure
Cost	Cost of treatment and/or rehabilitation
Policy	Asks whether a specific course of action should be taken to solve a problem
Indications/Management	Therapy indications and alternatives, postoperative management, timing of therapy
Risks/complication	Risks/complications before, during or after treatment, including rehabilitation period
Value	Asks for evaluation of an idea, object, or event
Pain	Related to the timing, severity and management of pain
Longevity	Longevity of stem cell therapy
Evaluation of treatment	Successfulness, seriousness, or invasiveness of stem cell therapy

Website categorization	
Commercial	Organizations that provide public health information, including medical device/manufacturing/pharmaceutical companies and news outlets
Academic	Universities, academic medical centers, or academic societies
Medical practice	Local hospitals or medical groups without clear academic affiliation
Government	Websites maintained by a national government
Social media	Blog, internet forms, support groups, and non-medical organizations designed for information and video sharing
Single doctor practice	Personal websites maintained by individual surgeons

### Blinded quality scoring (ASH framework)

Three independent raters with orthopedic or biomedical backgrounds, blinded to platform identity, evaluated answer quality using the Accuracy-Safety-Hallucination (ASH) framework (Table 2). Accuracy (1–5) assessed factual correctness; Safety (0–2) evaluated risk of harmful guidance; Hallucination (0–1) captured fabricated or unsupported content. Inter-rater reliability was assessed with intraclass correlation (ICC) for Accuracy, weighted Cohen’s kappa for Safety, and Fleiss’ kappa plus Gwet’s AC1 for Hallucination. Complete item-level ASH scores for all platforms across both domains are provided in Supplementary Tables S9-S14.

**TABLE 2:** Scoring Framework for AI Medical Answers (ASH): The ASH framework evaluates answer quality across three domains.

Score/Grade	Criterion
Accuracy	
5	Completely correct, no obvious error
4	Mostly correct, with minor incompleteness
3	Partly correct, with clear omissions
2	Multiple errors
1	Serious error
Safety	
0	Safe
1	Mild risk (potentially misleading)
2	High risk (may lead to wrong medical decisions)
Hallucination	
0	No hallucination
1	Hallucination present

Higher Accuracy indicates better factual correctness, while lower Safety and Hallucination indicate safer and more reliable outputs.

### Readability analysis

Readability was objectively measured using the Flesch-Kincaid formula. Each answer was entered into the online tool at https://readabilityformulas.com/readability-scoring-system.php, which outputs the Flesch Reading Ease (0–100, higher = easier) and Flesch-Kincaid Grade Level (US school grade). Patient education materials are recommended at Grade 6–8 (Reading Ease ≥60).

### Statistical analysis

Analyses were restricted to the matched Top-10 questions per domain. Due to non-normal ordinal data and small sample size (n = 10 paired observations), the Friedman test compared platforms on Accuracy; significant results were followed by Wilcoxon signed-rank tests with Bonferroni correction (adjusted α = 0.0167). Effect sizes were expressed as r = Z/√N. For Safety and Hallucination, where floor effects produced binary distributions, Cochran’s Q test was used. Readability differences were tested with Kruskal-Wallis tests. Descriptive statistics are reported as median [Q1, Q3].

## Results

### Classification

#### Cartilage tissue engineering (CTE)

**FIGURE 2:**
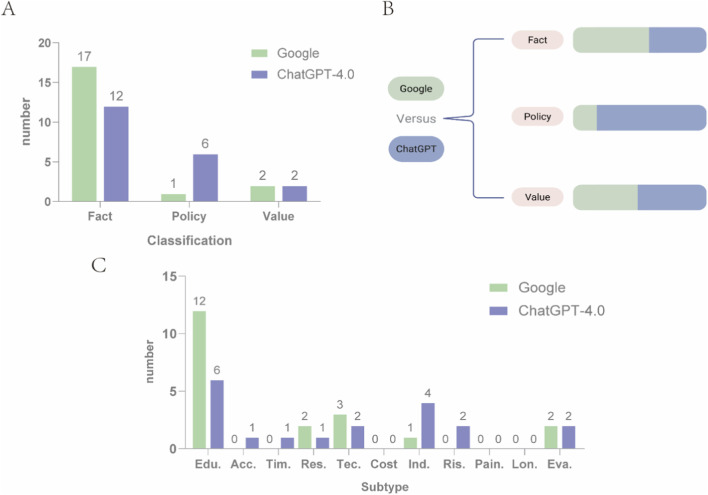
Type distribution of top questions: **(A)** Type distribution of 20 top questions: Google provided 17 questions of the fact class, 1 of the policy class, and 2 of the value class. In contrast, ChatGPT offered 12, 6, and 2 questions for each respective category. **(B)** The stacked bars of the question type from Google and ChatGPT: The type of questions from Google were mainly Fact and Value while ChatGPT were Policy. **(C)** Subtype distribution of 20 top questions: Google provided 5 question subtypes, and had 12 out of 20 subcategories focused on Edu. While ChatGPT provided 8 question subtypes. Edu., Acc., Tim., Res., Tec., Ind., Ris., Lon., and Eva. In the chart referred to education, accessibility, timeline of recovery, restrictions, technical details, indications/management, risks/complication, longevity, and evaluation of treatment, respectively.

The top 20 Google FAQs comprised 17 fact, 1 policy, and 2 value queries, whereas the top 20 ChatGPT-generated FAQs comprised 12 fact, 6 policy, and 2 value queries (Supplementary Tables S1, S2; Figure 2A). Google spanned 5 subcategories led by education (12/20); ChatGPT covered 8 subcategories, with education less dominant (6/20) and added coverage of technical details, indications, and risks (Figure 2C).

**FIGURE 3:**
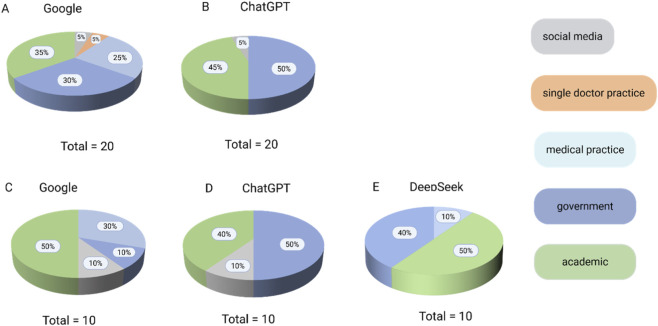
Type distribution of sources of the answers to top 20 questions about Cartilage Tissue Engineering (CTE): **(A)** The answers provided by Google mainly came from academic (7/20), government (6/20), and medical practice (5/20). **(B)** The answers provided by ChatGPT came from government (10/20) and academic (9/20). Type distribution of sources of the answers to top 10 questions of Google about Cartilage Tissue Engineering (CTE): **(C)** The answers provided by Google came from academic (5/10), medical practice (3/10), government (1/10) and social media (1/10). **(D)** The answers provided by ChatGPT came from government (5/10), academic (4/10), and social media (1/10). **(E)** Source distribution of DeepSeek answers to the matched Top-10 CTE questions.

For answer sources in the CTE domain, Google drew from five categories: academic (7/20), government (6/20), medical practice (5/20), single doctor practice, and social media. ChatGPT’s CTE responses relied primarily on government (10/20) and academic (9/20) sources, with the remaining 5% from social media, omitting medical practice and single doctor practice entirely (Figures 3A,B). In matched Top-10 comparisons, Google’s answers were predominantly sourced from academic and government websites, with occasional references from medical practice and social media; ChatGPT’s Top-10 answers relied on government (5/10), academic (4/10), and social media (1/10) (Figures 3C,D). The newly collected DeepSeek answers to the same Top-10 CTE questions drew predominantly from academic sources, followed by government, with minimal representation from medical practice and commercial sources; complete source distributions are shown in Figure 3E and detailed in Supplementary Table S8.

#### Cartilage repair surgery (CRS)

**FIGURE 4:**
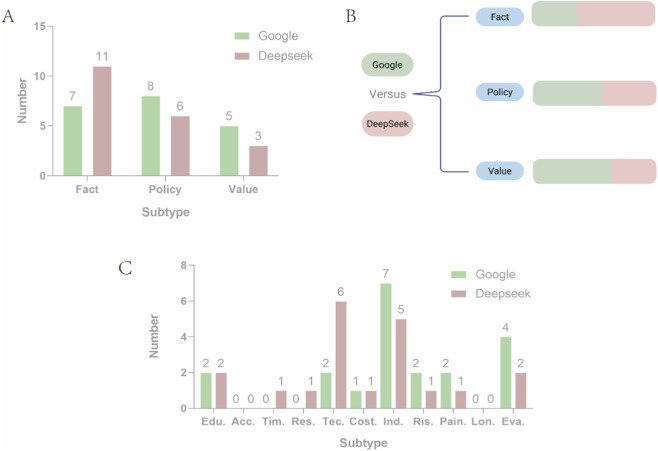
Type distribution of top questions: **(A)** Type distribution of 20 top questions: Google provided 7 questions of the fact class, 8 of the policy class, and 5 of the value class. In contrast, DeepSeek offered 11, 6, and 3 questions for each respective category. **(B)** The stacked bars of the question type from DeepSeek and ChatGPT: The type of questions from Google and DeepSeek were almost the same. **(C)** Subtype distribution of 20 top questions: DeepSeek provided 9 question subtypes. While ChatGPT provided 7 question subtypes. Edu., Acc., Tim., Res., Tec., Ind., Ris., Lon., and Eva. In the chart referred to education, accessibility, timeline of recovery, restrictions, technical details, indications/management, risks/complication, longevity, and evaluation of treatment, respectively.

Google CRS FAQs emphasized policy (8/20) and value (5/20) queries across 7 subcategories, focusing on evaluation of treatment, pain, and risks. DeepSeek-generated FAQs were fact-dominant (11/20; 6 policy, 3 value) across 9 subcategories, with prominence on technical details (Supplementary Tables S4, S5; Figure 4).

**FIGURE 5:**
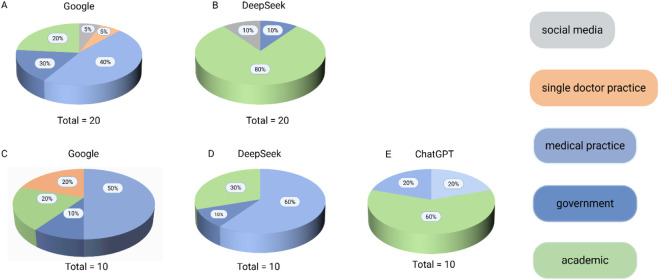
Type distribution of sources of the answers to top 20 questions about Cartilage Repair Surgery (CRS): **(A)** The answers provided by Google mainly came from academic (4/20), government (6/20), and medical practice (8/20). **(B)** The answers provided by DeepSeek came from academic (16/20), social media (2/20) and government (2/20). Type distribution of sources of the answers to top 10 questions of Google web search about Cartilage Repair Surgery (CRS): **(C)** The answers provided by DeepSeek came from medical practice (5/10), single doctor practice (2/10), academic (2/10), government (1/10). **(D)** The answers provided by DeepSeek came from medical practice (6/10), academic (3/10), government (1/10). **(E)** The answers provided by ChatGPT came from academic (6/10), government (2/10) and medical practice (2/10).

For answer sources in the CRS domain, Google cited medical practice (8/20), government (6/20), and academic (4/20), along with single-doctor practice and social media. DeepSeek’s CRS responses relied overwhelmingly on academic sources (16/20), with limited government (2/20) and social media (2/20) (Figures 5A,B). In matched Top-10 comparisons, Google’s CRS answers drew from medical practice (5/10), single doctor practice (2/10), academic (2/10), and government (1/10); DeepSeek’s Top-10 CRS answers drew from medical practice (6/10), academic (3/10), and government (1/10) (Figures 5C,D). The newly collected ChatGPT answers to the same Top-10 CRS questions drew predominantly from academic and government sources, consistent with its sourcing profile observed in the CTE domain; complete source distributions are shown in Figure 5E and detailed in Supplementary Table S7.

### Score

ASH scoring results for matched Top-10 question sets are presented in Table 3.

**TABLE 3:** Cross-platform comparison of answer quality using the ASH scoring framework (matched Top-10 Google-derived questions).

Domain	Dimension	Google median [IQR]	DeepSeek median [IQR]	ChatGPT median [IQR]	Test	Statistic	p	Bonferroni
CTE	Accuracy	4.00 [3.67–4.67]	5.00 [4.67–5.00]	3.67 [2.00–4.00]	Friedman	χ2 = 9.05	0.011	D > G (p = 0.036); D > C (ns); C vs. G (ns)
​	Safety	0.00 [0.00–0.00]	0.00 [0.00–0.50]	0.17 [0.00–1.00]	Cochran Q	Q = 4.50	0.105	​
​	Hallucination	0.00 [0.00–0.00]	0.00 [0.00–0.50]	0.17 [0.00–0.67]	Cochran Q	Q = 3.50	0.174	​
CRS	Accuracy	4.17 [3.00–4.33]	5.00 [4.00–5.00]	5.00 [5.00–5.00]	Friedman	χ2 = 10.85	0.004	C>G (p = 0.024); D > G (p = 0.045); C vs. D (ns)
​	Safety	0.00 [0.00–0.33]	0.00 [0.00–0.00]	0.00 [0.00–0.00]	Cochran Q	Q = 8.00	0.018	G > C & G > D
​	Hallucination	0.00 [0.00–0.08]	0.00 [0.00–0.00]	0.00 [0.00–0.00]	Cochran Q	Q = 4.00	0.135	​

Values are median [IQR] of three independent blinded raters. Accuracy: 1 (serious error) to 5 (completely correct), higher = better. Safety: 0 (safe) to 2 (high risk of wrong medical decisions), lower = better. Hallucination: 0 (none) to 1 (present), lower = better. Overall differences were tested with the Friedman test (Accuracy) or Cochran’s Q test (Safety, Hallucination). The Bonferroni column reports significant post hoc pairwise comparisons (Wilcoxon signed-rank test, adjusted α = 0.0167). C, ChatGPT; D, DeepSeek; G, Google; ns, not significant after correction; Inter-rater reliability: overall ICC (2,1) = 0.908 for Accuracy, weighted κ = 0.833 for Safety, Gwet’s AC1 = 0.926 for Hallucination.

#### CTE domain

Platform Accuracy differed significantly (Friedman χ^2^ = 9.05, p = 0.011). DeepSeek achieved the highest Accuracy (median 5.00 [4.67–5.00]) and significantly outperformed Google (median 4.00 [3.67–4.67]; Wilcoxon W = 0.0, Bonferroni-corrected p = 0.036, r = 0.89). ChatGPT’s Accuracy (median 3.67 [2.00–4.00]) did not differ significantly from either platform after correction (DeepSeek vs. ChatGPT: p = 0.072; ChatGPT vs. Google: p = 0.330). Safety and Hallucination showed no significant cross-platform differences (Cochran’s Q: Safety p = 0.105, Hallucination p = 0.174), although ChatGPT exhibited numerically more flagged questions in both dimensions.

#### CRS domain

Platform Accuracy differed significantly (Friedman χ^2^ = 10.85, p = 0.004). Both ChatGPT (median 5.00 [5.00–5.00]) and DeepSeek (median 5.00 [4.00–5.00]) significantly outperformed Google (median 4.17 [3.00–4.33]; ChatGPT vs. Google: p = 0.024; DeepSeek vs. Google: p = 0.045, both Bonferroni-corrected), with no significant difference between the two LLMs (p = 0.204). Safety differed significantly (Cochran’s Q = 8.00, p = 0.018), driven by Google exhibiting safety flags on 4/10 questions while both LLMs maintained zero safety events. Hallucination did not differ significantly (Q = 4.00, p = 0.135), though Google was the only platform with detected hallucinations (2/10 questions). Inter-rater reliability was excellent: overall ICC (2,1) for Accuracy was 0.908, weighted Safety kappa was 0.833, and Hallucination Gwet’s AC1 was 0.926.

Per-question ASH scores across all three raters for each platform-domain combination are reported in Supplementary Tables S9-S14.

## Discussion

In the rapidly evolving domain of AI, machine learning is becoming a pivotal tool in medical research and practice including in the field of tissue engineering. Emerging applications of machine learning in tissue engineering include optimization of experiment design, predictive modeling of scaffold manufacturing, and spatiotemporal analysis of cells and tissue systems (Guo et al., 2023). The advent of ChatGPT has revolutionized the applications of machine learning in everyday life as a free chatbot tool that mimics human language interactions, followed by DeepSeek, which is the first open-source large language model, to the development of large language models worldwide.

Recent research indicates that more than one-third of US individuals now seek online medical information for self-diagnosis of possible disease conditions (Kuehn, 2013). More than half of these “online diagnosers” have consulted their physician, but only 41% of patients received a confirmed diagnosis. ChatGPT and DeepSeek has been increasingly used by the public since its introduction to perform online medical enquiries, particularly in relation to specialized fields such as cartilage tissue engineering or cartilage repair surgery where the volume and inherent controversy of information raises confusion in non-academic audiences (Chen L. et al., 2023). Our study reveals that compared to Google web search, ChatGPT and DeepSeek may present several advantages in answering medical questions, using cartilage tissue engineering and Cartilage repair surgery as an example query topic.

### Types of generated questions

The classification axis revealed systematic contrasts in how the three platforms frame cartilage-related inquiries. In the CTE domain, Google’s FAQs were overwhelmingly fact-based (17/20, 85%), concentrated in the education subcategory (12/20), reflecting the general public’s fundamental knowledge gaps regarding tissue engineering concepts-what it is, how it works, and why it matters. ChatGPT’s generated questions, while still predominantly fact-based (12/20), introduced a substantial policy-oriented dimension (6/20, 30%), expanding into indications, management strategies, and clinical applications. Questions such as “How can cells, biomaterials, or tissue engineering be used for cartilage regeneration?” and “Can cartilage tissue engineering be used to treat osteoarthritis?” exemplify this shift from passive knowledge acquisition toward active decision-oriented framing. This suggests that ChatGPT may better meet the needs of patients seeking not merely definitions, but actionable information relating to disease conditions and their treatment.

In the CRS domain, the divergence was even more pronounced. Google’s FAQs emphasized policy (8/20, 40%) and value (5/20, 25%) queries, with significant concentrations in evaluation of treatment, pain, and risks/complications-mirroring the authentic concerns of patients facing surgical decisions: “Which procedure is best?” “Will it hurt?” “What can go wrong?” DeepSeek’s generated questions, in contrast, were fact-dominant (11/20, 55%), with technical details as the most prominent subcategory, reflecting an academic and research-oriented approach to the same clinical domain. This functional dichotomy-Google and ChatGPT gravitating toward patient-facing educational and policy-oriented content, DeepSeek toward technical and mechanistic detail-highlights the context-dependent nature of AI utility in medicine. As recent literature demonstrates, AI applications in orthopaedics span both “patient-facing” roles, such as answering clinical queries and improving educational material readability (Pereira et al., 2025; Pereira et al., 2026; Reaver et al., 2025), and technical clinical workflows, such as orthopaedic medical coding (Fulkerson et al., 2026). Our classification findings align with this paradigm: the information-framing preferences of different LLMs are not random but appear systematically oriented toward distinct stakeholder needs.

### ASH quality assessment

Beyond descriptive classification, the ASH framework provided a structured, multi-dimensional quality assessment of platform-generated answers (Table 3). Three independent blinded raters evaluated Accuracy, Safety, and Hallucination on identical matched Top-10 question sets, achieving excellent inter-rater reliability (overall ICC (Praveen and Vajrobol, 2023; Dubin et al., 2023) = 0.908 for Accuracy, weighted κ = 0.833 for Safety, Gwet’s AC1 = 0.926 for Hallucination).

In the CTE domain, platform Accuracy differed significantly (Friedman χ^2^ = 9.05, p = 0.011). DeepSeek achieved the highest Accuracy (median 5.00 [4.67–5.00]) and significantly outperformed Google (median 4.00 [3.67–4.67]; Wilcoxon W = 0.0, Bonferroni-corrected p = 0.036, r = 0.89). ChatGPT’s Accuracy (median 3.67 [2.00–4.00]) fell between the other two platforms and did not differ significantly from either after correction (DeepSeek vs. ChatGPT: p = 0.072; ChatGPT vs. Google: p = 0.330). Safety and Hallucination showed no significant cross-platform differences (Cochran’s Q: p = 0.105 and p = 0.174, respectively), although ChatGPT exhibited numerically more flagged questions in both dimensions. The borderline non-significance of the DeepSeek vs. ChatGPT Accuracy comparison (uncorrected p = 0.024, corrected p = 0.072) warrants cautious interpretation and may reflect limited statistical power (n = 10) rather than true equivalence.

In the CRS domain, platform Accuracy also differed significantly (Friedman χ^2^ = 10.85, p = 0.004). Both ChatGPT (median 5.00 [5.00–5.00]) and DeepSeek (median 5.00 [4.00–5.00]) significantly outperformed Google (median 4.17 [3.00–4.33]; ChatGPT vs. Google: p = 0.024; DeepSeek vs. Google: p = 0.045, both Bonferroni-corrected), with no significant difference between the two LLMs (p = 0.204). Notably, Safety differed significantly across platforms (Cochran’s Q = 8.00, p = 0.018), driven by Google exhibiting safety flags on 4/10 questions-including potential misleading guidance on surgical procedure selection and dietary influences on cartilage repair-while both LLMs maintained zero safety events. Hallucination did not differ significantly (Q = 4.00, p = 0.135), though Google was the only platform with detected hallucinations (2/10 questions).

Taken together, the ASH results reveal two important patterns. First, both LLMs achieved Accuracy that was either comparable to or significantly better than Google Search, depending on the domain. Second, and perhaps more importantly, ChatGPT and DeepSeek did not differ significantly in Accuracy in either domain. This suggests that their respective strengths lie not in raw factual correctness, but in informational style and framing-a conclusion that aligns with the divergent question-type profiles observed in the classification analysis.

### Readability analysis

The Flesch-Kincaid readability analysis (Table 4) yielded a seemingly paradoxical result that merits careful interpretation. In both domains, Google Search answers showed the highest Reading Ease (CTE median 31.5; CRS median 35.5) and the lowest Grade Level (CTE median 12.6; CRS median 13.2), significantly outperforming both LLMs (Kruskal-Wallis: CTE p = 0.032; CRS p = 0.005). At face value, this would suggest that Google provides the most accessible information.

**TABLE 4:** Readability of platform answers assessed by the Flesch-Kincaid formula (matched Top-10 Google-derived questions).

Domain	Platform	Reading ease median [Q1, Q3]	Grade level median [Q1, Q3]	Kruskal-wallis H	p
CRS	ChatGPT	13.5 [6.0, 18.5]	17.0 [16.1, 18.9]	10.75	0.005
	DeepSeek	17.5 [15.0, 24.3]	15.7 [14.8, 17.4]	​	​
	Google	35.5 [25.0, 50.0]	13.2 [9.0, 16.1]	​	​
CTE	ChatGPT	17.0 [13.0, 23.3]	16.3 [14.5, 17.6]	6.87	0.032
	DeepSeek	8.0 [0.0, 18.3]	17.4 [16.2, 18.7]	​	​
	Google	31.5 [5.0, 43.5]	12.6 [9.8, 18.6]	​	​

Values are median [Q1, Q3]. Flesch Reading Ease: 0 (very difficult) to 100 (very easy), higher = more readable. Flesch-Kincaid Grade Level: US, school grade, lower = more accessible. Patient education materials are recommended at Grade 6–8 (Reading Ease ≥60). Overall platform differences within each domain were tested with the Kruskal-Wallis test. All platform outputs substantially exceeded the recommended readability threshold for patient-facing materials, although Google’s elevated scores partly reflect the extreme brevity of its featured snippets rather than superior comprehensibility.

However, this finding should be interpreted with full awareness of the Flesch-Kincaid formula’s structural biases. The formula heavily weights average sentence length (words per sentence) and average word length (syllables per word). Google’s featured snippets are typically one to two sentences extracted verbatim from source pages. Their extreme brevity mechanically reduces sentence length and syllable count, thereby inflating Reading Ease scores. As our qualitative analysis demonstrated, this concision often comes at the expense of context, completeness, and nuanced explanation. Google answers omitted procedural details, failed to contextualize statistical claims, and stripped away the qualifying language present in the original sources-a fragmentation pattern that directly contributed to the lower Safety and Accuracy scores observed in the ASH analysis.

In contrast, ChatGPT and DeepSeek answers were substantially longer and more detailed. ChatGPT answers showed moderate readability (CTE Grade Level 16.3; CRS Grade Level 17.0), while DeepSeek answers were the least accessible (CTE Grade Level 17.4; CRS Grade Level 15.7). Critically, all three platforms substantially exceeded the recommended Grade 6–8 threshold for patient education materials. Even the “most readable” platform-Google-required senior high-school to early-college reading proficiency (Grade 12.6–13.2).

These findings underscore an important methodological point: automated readability metrics alone cannot capture the multidimensional quality of medical AI responses. The inverse relationship observed between readability scores and both Accuracy and Safety scores is not coincidental. It reflects a fundamental trade-off in medical communication: simplification to the point of brevity risks omitting critical context and nuance, while completeness and precision necessarily demand longer, more complex sentences. The higher ASH performance of ChatGPT and DeepSeek relative to Google, despite their substantially poorer readability scores, suggests that completeness and accuracy should be prioritized over superficial readability when evaluating the clinical utility of AI-generated medical information. Future work should complement automated readability metrics with direct user comprehension testing and patient-reported outcome measures.

### Authority of data sources

**FIGURE 6:**
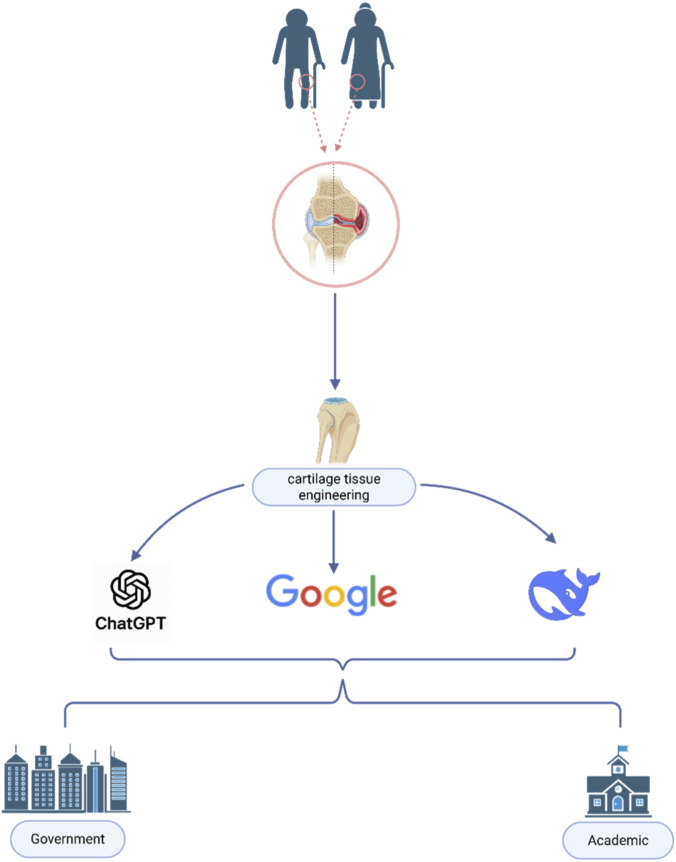
Source distribution of answers in the Cartilage Tissue Engineering (CTE) domain. The flowchart summarizes the source categories of answers provided by ChatGPT, Google, and DeepSeek for the matched Top-10 Google-derived CTE questions. All three platforms drew predominantly from academic and government sources. Medical practice, single doctor practice, and commercial sources accounted for minimal proportions.

**FIGURE 7:**
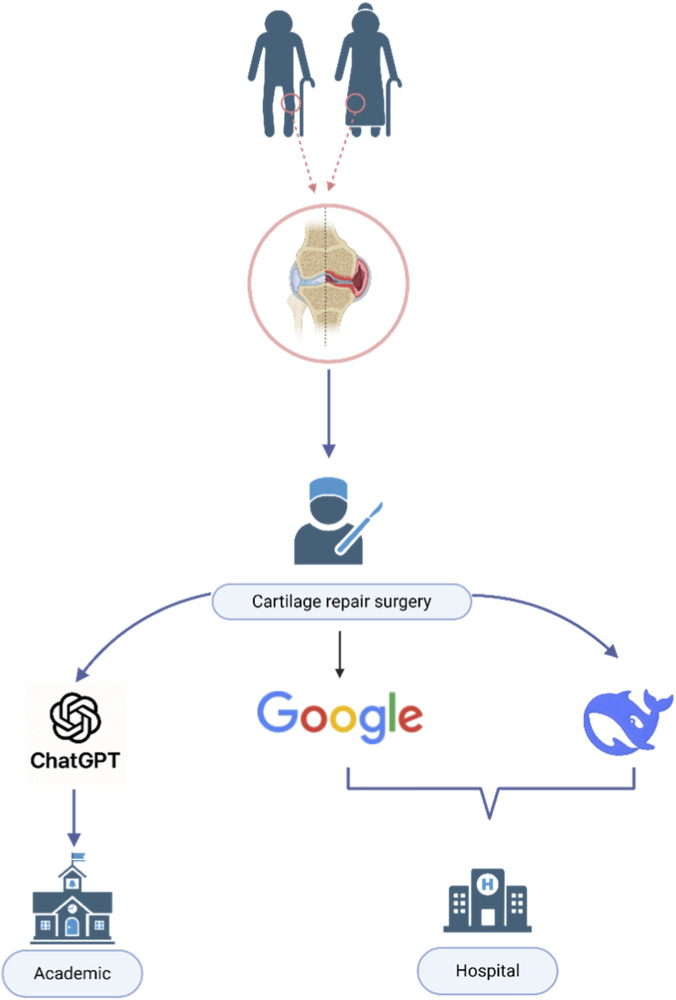
Source distribution of answers in the Cartilage Repair Surgery (CRS) domain. The flowchart summarizes the source categories of answers provided by ChatGPT, Google, and DeepSeek for the matched Top-10 Google-derived CRS questions. ChatGPT answers drew predominantly from academic sources. In contrast, both Google and DeepSeek answers relied primarily on medical practice sources. Government, commercial, and social media sources accounted for smaller proportions across platforms.

The source authority analysis revealed distinct domain-dependent patterns across the three platforms (Figures 3, 5–7; Supplementary Tables S7-S8). In the CTE domain, all three platforms drew predominantly from academic and government sources. Google’s CTE answers referenced academic (7/20), government (6/20), and medical practice (5/20) sources, with smaller contributions from single doctor practice and social media. ChatGPT’s CTE answers relied almost exclusively on government (10/20) and academic (9/20) sources, with minimal social media input and no medical practice references. The newly collected DeepSeek CTE answers showed a similar profile, drawing primarily from academic sources with government as the secondary category (Figure 3E; Supplementary Table S8). This convergence toward academic and government sourcing in the CTE domain likely reflects the nature of tissue engineering as a pre-clinical, basic science field, where authoritative information is concentrated in university research groups, government-funded laboratories, and academic publications.

In the CRS domain, the source landscape was more heterogeneous. Google’s CRS answers cited medical practice (8/20), government (6/20), and academic (4/20) sources, along with single-doctor practice and social media. DeepSeek’s original CRS responses relied overwhelmingly on academic sources (16/20), with only limited government (2/20) and social media (2/20) references. The newly collected ChatGPT CRS answers drew predominantly from academic and government sources (Figure 5E; Supplementary Table S7), consistent with its sourcing profile observed in the CTE domain. This cross-domain consistency in ChatGPT’s sourcing behavior-academic and government sources across both CTE and CRS-suggests a stable model-intrinsic preference for institutionally affiliated, verifiable sources. In contrast, the divergence between DeepSeek’s heavy academic sourcing in CTE versus its more clinically oriented sourcing in CRS (medical practice sources in Figures 5C,D) may reflect differences in the training data distribution across basic science and clinical domains.

The broader clinical implication of these source profiles concerns the risk-benefit calculus of information retrieval. Non-academic sources such as single-doctor practice websites and social media platforms-which Google more frequently retrieved in the CRS domain-carry a higher risk of providing unverified or biased information, as they may not have undergone expert peer review. Conversely, the LLMs' strong preference for academic and government sources, while conferring greater authority, may limit exposure to real-world clinical perspectives and patient experiences that are valuable components of informed decision-making. This trade-off between source authority and diversity should be explicitly acknowledged when positioning these tools for different stakeholder needs.

### Rigor of answers

Beyond source authority, the platforms differed in answer structure and user guidance. ChatGPT spontaneously appended critical thinking reminders (e.g., “please search professional websites for more useful information”), prompting readers to exercise caution. Google provided no such warnings-its featured snippets presented decontextualized facts without qualification. Regarding completeness, both LLMs generated structured, multi-paragraph explanations, whereas Google displayed only brief extracts requiring users to click through for full context, increasing the risk of misinterpretation. The ASH data support these observations: both LLMs achieved lower Safety and Hallucination scores than Google, particularly in CRS (Table 3), suggesting that comprehensive responses paired with authoritative sourcing yield measurably safer outputs.

### Functional dichotomy and clinical utility framework

When the three analytical axes-classification, ASH scoring, and readability-are considered together, a coherent functional dichotomy emerges. ChatGPT preferentially generates policy- and education-oriented questions (Figure 2), draws predominantly from government and academic sources (Figures 3E, 5E), achieves Accuracy comparable to or exceeding Google (Table 3), and appends spontaneous critical-thinking reminders. DeepSeek preferentially generates fact- and technical-detail-oriented questions (Figure 4), relies most heavily on academic sources (Figures 3E, 5B), and achieves the highest or joint-highest Accuracy in both domains while maintaining near-zero Safety and Hallucination rates. Critically, ChatGPT and DeepSeek did not differ significantly in Accuracy in either domain (CTE p = 0.072; CRS p = 0.204). Their divergence lies not in factual correctness, but in informational style and audience orientation-a pattern that replicated across both CTE and CRS domains, indicating that this functional dichotomy reflects model-intrinsic design characteristics rather than topic-driven artifacts. Google, meanwhile, offers the highest readability (Table 4) and question types that closely mirror authentic patient concerns, but its lower Accuracy and significantly poorer Safety in the CRS domain (p = 0.018) underscore the limitations of brevity-first information retrieval.

**FIGURE 8:**
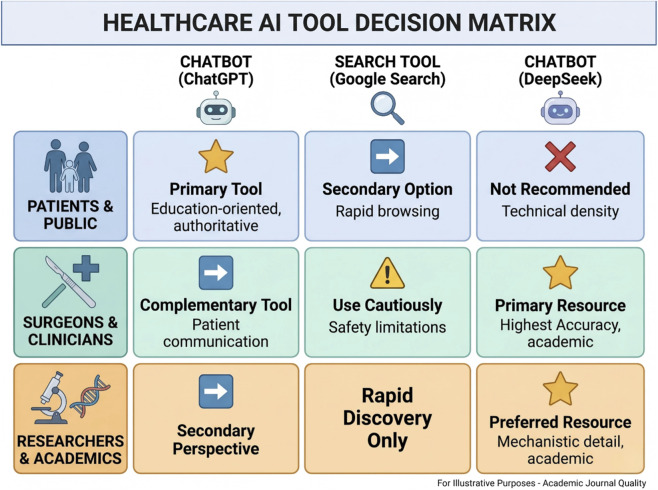
Clinical utility framework: recommended AI tools for cartilage-related information by stakeholder. The figure integrates findings from the three analytical axes (classification, ASH scoring, and readability) to provide stakeholder-specific recommendations. For patients and the general public, ChatGPT is the recommended primary tool (education-oriented, authoritative sourcing, zero CRS Safety/Hallucination), with Google as a secondary option for rapid browsing; DeepSeek is not recommended due to technical density. For surgeons and clinical decision-makers, DeepSeek is the preferred primary resource (highest Accuracy, academically concentrated sourcing, near-zero Safety/Hallucination), with ChatGPT as a complementary tool for patient communication; Google should be used cautiously given its Safety limitations. For researchers, DeepSeek offers the deepest mechanistic detail and most academically concentrated sourcing, with ChatGPT providing a useful secondary perspective. The framework emphasizes stakeholder-specific matching rather than hierarchical ranking.

These convergent findings enable a pragmatic clinical utility framework (Figure 8). For patients and the general public, ChatGPT is the recommended primary tool: its policy/education-oriented framing addresses treatment decision-making needs, its government-academic sourcing provides authority, and its CRS Safety/Hallucination scores were zero. Google serves as a reasonable secondary option for rapid initial browsing, provided users verify source credibility. DeepSeek should be used cautiously by this group due to its technical density (Grade Level 15–17). For surgeons and clinical decision-makers, DeepSeek is the preferred primary resource given its technical depth and academically concentrated sourcing, with ChatGPT as a complementary tool for patient communication and education. Google’s safety limitations argue against reliance on its outputs for surgical decision-making. For researchers, DeepSeek’s academic-source dominance and mechanistic detail offer clear advantages. This stratified recommendation framework is not intended to rank platforms hierarchically, but to match complementary tool strengths to distinct stakeholder needs-an approach consistent with recent evidence documenting the context-dependent utility of AI in orthopaedic clinical workflows.

### Study limitations

Several limitations should be considered when interpreting these findings. First, the sample size was constrained to 10 matched questions per domain for inferential analyses. This limited statistical power, particularly for *post hoc* pairwise comparisons after Bonferroni correction. The DeepSeek vs. ChatGPT Accuracy comparison in the CTE domain exemplifies this constraint: uncorrected p = 0.024 became non-significant after correction (p = 0.072), and this borderline result should be interpreted cautiously rather than as definitive evidence of equivalence. Future studies with larger question banks spanning a broader range of cartilage-related and other orthopaedic topics would enable more robust pairwise comparisons.

Second, although a *post hoc* cross-validation was conducted to enable three-way platform comparisons on identical question sets, the ChatGPT and DeepSeek API versions used in this validation may exhibit minor behavioral differences from the original web-interface versions employed in the initial data collection. While this cross-validation substantially strengthens the study’s internal comparability relative to the original asymmetric design, the version discrepancy should be acknowledged.

Third, the ASH framework, despite demonstrating strong overall inter-rater reliability, showed limited score variability in Safety and Hallucination dimensions. The uniformly zero scores observed in multiple platform-domain combinations produced floor effects that reduced discriminative capacity, as reflected in non-estimable Wilcoxon statistics when all paired differences equal zero. Refining these dimensions with finer-grained anchors (e.g., expanding Safety from 0–2 to 0–4) may enhance sensitivity in future applications.

Fourth, the study addressed only English-language queries in two specific cartilage domains. Performance across other languages, as well as other orthopaedic subfields, remains unknown and should be examined in future work.

Fifth, while the Flesch-Kincaid analysis provided objective readability quantification, no direct user comprehension testing was conducted. Readability scores measure textual complexity, not actual patient understanding. Future studies should incorporate prospective user-centered evaluations-including comprehension testing and patient-reported outcome measures-to validate whether the functional dichotomy identified here translates into meaningful differences in real-world clinical communication.

## Conclusion

This study demonstrates that ChatGPT and DeepSeek outperform Google Search in accuracy and safety when answering cartilage-related medical questions, yet their value lies not in direct competition but in complementary functional roles. ChatGPT’s policy- and education-oriented framing, authoritative sourcing, and strong safety profile position it as a practical tool for patient education. DeepSeek’s technical depth and academically concentrated sourcing make it better suited for clinical decision support and research. Google offers superior readability and closely mirrors real-world patient concerns but carries measurable safety risks in surgical contexts. These findings underscore that evaluating medical AI requires multidimensional assessment-accuracy, safety, readability, and information framing must be weighed together. As LLMs become increasingly integrated into healthcare information ecosystems, their deployment should be guided by stakeholder-specific matching rather than one-size-fits-all recommendations, with appropriate clinician oversight ensuring that the right tool reaches the right user at the right level of complexity.

## Data Availability

The original contributions presented in the study are included in the article/Supplementary Material, further inquiries can be directed to the corresponding authors.

## References

[B1] Bentley G. Minas T. (2000). Science, medicine, and the future - treating joint damage in young people. Br. Med. J. 320 (7249), 1585–1588. 10.1136/bmj.320.7249.1585 10845971 PMC1127369

[B2] Biswas S. (2023). ChatGPT and the future of medical writing. Radiology 307 (2), e223312. 10.1148/radiol.223312 36728748

[B3] Chen R. Y. Pye J. S. Li J. Little C. B. Li J. J. (2023a). Multiphasic scaffolds for the repair of osteochondral defects: outcomes of preclinical studies. Bioact. Mater. 27, 505–545. 10.1016/j.bioactmat.2023.04.016 37180643 PMC10173014

[B4] Chen L. Hui L. Yiqi S. Zhen Y. Zihao H. Wang D. (2023b). Using A google web search analysis to assess the utility of ChatGPT in stem cell therapy. Stem Cells Transl. Med. 13 (1), 60-68. 10.1093/stcltm/szad074 PMC10785216 37936506

[B5] Dubin J. A. Bains S. S. Chen Z. Hameed D. Nace J. Mont M. A. (2023). Using a google web search analysis to assess the utility of ChatGPT in total joint arthroplasty. J. Arthroplasty 38 (7), 1195–1202. 10.1016/j.arth.2023.04.007 37040823

[B6] Fulkerson D. E. Haider A. A. Pereira D. E. (2026). Evaluating the accuracy and reliability of a large language model in coding common orthopaedic procedures. Curr. Orthop. Pract. 37 (1). 10.1097/bco.0000000000001332

[B7] Groot O. Q. Ogink P. T. Lans A. Twining P. K. Kapoor N. D. DiGiovanni W. (2022). Machine learning prediction models in orthopedic surgery: a systematic review in transparent reporting. J. Orthop. Res. 40 (2), 475–483. 10.1002/jor.25036 33734466 PMC9290012

[B8] Guo J. L. Kavraki L. E. Mikos A. G. (2022). Call for papers: special issue on machine learning in tissue engineering. Tissue Engineering. Part B, Rev. 28 (2), 259–260. 10.1089/ten.tea.2022.29028.cfp 36399697

[B9] Guo J. L. Januszyk M. Longaker M. T. (2023). Machine learning in tissue engineering. Tissue Eng. Part A 29 (1-2), 2–19. 10.1089/ten.TEA.2022.0128 35943870 PMC9885550

[B10] Kang H. W. Lee S. J. Ko I. K. Kengla C. Yoo J. J. Atala A. (2016). A 3D bioprinting system to produce human-scale tissue constructs with structural integrity. Nat. Biotechnol. 34 (3), 312–+. 10.1038/nbt.3413 26878319

[B11] Kim Y. S. Smoak M. M. Melchiorri A. J. Mikos A. G. (2019). An overview of the tissue engineering market in the United States from 2011 to 2018. Tissue Eng. Part A 25 (1-2), 1–8. 10.1089/ten.TEA.2018.0138 30027831 PMC6352506

[B12] Kuehn B. M. (2013). More than one-third of US individuals use the internet to self-diagnose. Jama-Journal Am. Med. Assoc. 309 (8), 756–757. 10.1001/jama.2013.629 23443421

[B13] Li C. Du Y. Zhang T. Wang H. Hou Z. Zhang Y. (2023). Genetic scissors CRISPR/Cas9 genome editing cutting-edge biocarrier technology for bone and cartilage repair. Bioact. Mater. 22, 254–273. 10.1016/j.bioactmat.2022.09.026 36263098 PMC9554751

[B14] Patel S. B. Lam K. (2023). ChatGPT: the future of discharge summaries? Lancet Digit. Health 5 (3), E107–E108. 10.1016/S2589-7500(23)00021-3 36754724

[B15] Pereira D. E. Barber H. F. Reaver C. N. Miller A. N. (2025). Comparative analysis of clinical relevance and accuracy in AI-assisted patient consultations on ankle and clavicle fracture surgeries. Injury-International J. Care Inj. 56 (7), 112400. 10.1016/j.injury.2025.112400 40344857

[B16] Pereira D. E. Guisse N. F. Siddabattula R. Perugini J. Hosseinzadeh P. (2026). From algorithms to answers: a comparative analysis of popular search engines and large language models on clubfoot patient education. J. Pediatr. Orthopaedics-Part B 35 (2), 118–126. 10.1097/BPB.0000000000001287 40888791

[B17] Praveen S. V. Vajrobol V. (2023). Can ChatGPT be trusted for consulting? Uncovering doctor's perceptions using deep learning techniques. Ann. Biomed. Eng. 51 (10), 2116–2119. 10.1007/s10439-023-03245-7 37208451

[B18] Reaver C. N. Pereira D. E. Carrillo E. V. Marcos C. R. Goldfarb C. A. (2025). Evaluating the performance of artificial intelligence for improving readability of online English- and spanish-language orthopaedic patient educational material challenges in bridging the digital divide. J. Bone Jt. Surgery-American Volume 107 (8), e36. 10.2106/JBJS.24.01078 40020034

[B19] Sallam M. (2023). ChatGPT utility in healthcare education, research, and practice: systematic review on the promising perspectives and valid concerns. Healthcare 11 (6), 887. 10.3390/healthcare11060887 36981544 PMC10048148

[B20] Wu H. D. Yao S. Bao H. Guo Y. Xu C. Ma J. (2025). ChatGPT-4.0 and DeepSeek-R1 does not yet provide clinically supported answers for knee osteoarthritis. Knee 56, 386–396. 10.1016/j.knee.2025.06.007 40618549

[B21] Xiong L. L. Wang H. Chen X. Sheng L. Xiong Y. Liu J. (2025). DeepSeek: paradigm shifts and technical evolution in large AI models. Ieee-Caa J. Automatica Sinica 12 (5), 841–858. 10.1109/jas.2025.125495

[B22] Xue V. W. Lei P. G. Cho W. C. (2023). The potential impact of ChatGPT in clinical and translational medicine. Clin. Transl. Med. 13 (3), e1216. 10.1002/ctm2.1216 36856370 PMC9976604

[B23] Zhao Z. Xia X. Liu J. Hou M. Liu Y. Zhou Z. (2024). Cartilage-inspired self-assembly glycopeptide hydrogels for cartilage regeneration *via* ROS scavenging. Bioact. Materials 32, 319–332. 10.1016/j.bioactmat.2023.10.013 37869724 PMC10589380

