# Correction: AI dialogues in cartilage repair: which guides evidence-based decisions better?

**DOI:** 10.3389/fcell.2026.1900251

**Published:** 2026-06-22

**Authors:** Senyang Xiao, Hui Li, Dan Xing, Jiao Jiao Li

**Affiliations:** 1 Peking University People’s Hospital, Peking University, Beijing, China; 2 School of Biomedical Engineering, Faculty of Engineering & IT, University of Technology Sydney, Sydney, NSW, Australia

**Keywords:** cartilage surgery, cartilage tissue engineering, ChatGPT, deepseek, machine learning

Author Senyang Xiao was erroneously spelled as Sen Yang Xiao. An incorrect number was provided for National Key Research and Development Program of China. The correct number is 2024YFC2510400.

The original article has been updated.

