# Supplementary material for: AI dialogues in cartilage repair: which guides evidence-based decisions better?
Source: Front Cell Dev Biol. 2026 May 29;14:1768270. doi: 10.3389/fcell.2026.1768270 (PMC13260151; doi:10.3389/fcell.2026.1768270)
Supplement: Supplementary file 2 [file Table1.docx]

| **Supplementary Table 1.** Top 20 Q&As from Google Web Search | | |
| --- | --- | --- |
|  | question | Answer |
| 1 | What is the tissue engineering approach  for cartilage? (fact, education) | The principal concept of cartilage tissue engineering is to seed chondrocytes or chondroprogenitor cells within a three-dimensional biomaterial scaffold which is then cultured in a bioreactor to produce functional cartilage in vitro, which is then implanted in vivo. (academic) |
| 2 | What are the challenges of cartilage tissue  engineering? (fact, restriction) | Three major challenges in cartilage tissue engineering: poor integration, inflammation and phenotypic instability. Many traditional procedures, including surgical methods such as microfracture of subchondral bone and soft tissue transplantation, have been widely used to treat damaged cartilage. (academic) |
| 3 | What biomaterials are used in cartilage  tissue engineering? (fact, education) | Collagen-based matrices or collagen sponges are among the mostly used matrices for cartilage engineering. Collagen is naturally degraded by collagenases and serines proteases. Its degradation is controlled locally by the cells present in the tissue. (government) |
| 4 | How cells biomaterial or tissue engineering  can be used for cartilage regeneration? (policy, indications) | Biomaterials are used for promoting cartilage repair by providing scaffolds for cell attachment, growth and differentiation and could act as vehicles for protein and gene delivery to regenerate functional tissue. (academic) |
| 5 | Why is cartilage tissue engineering important? (fact, education) | Many people around the world suffer from cartilage defects, and in that sense, every step toward the repair of this tissue is very important. The cartilage scaffolds mimic ECM structure suitable for the seeding and cultivation of chondrocytes. (academic) |
| 6 | What is the technology for cartilage replacement? (fact, technical details) | Osetochondral grafting is a cartilage regeneration procedure that  replaces not only the cartilage but the underlying bone as well. It is often used when the cartilage is worn down to the bone. (medical practice) |
| 7 | Why can't we repair cartilage? (fact, restrictions) | Because cartilage does not have a blood supply, it has limited ability to repair itself. Cartilage regeneration, along with strengthening muscles around the joint, can help some patients delay joint replacement surgery for damaged joints.  (medical practice) |
| 8 | What is the most difficult tissue to engineer? (fact, education) | Cartilage is a difficult tissue to engineer as cells often tend to continue differentiation toward bone. (academic) |
| 9 | Why is cartilage so hard to repair? (fact, education) | Because articular cartilage doesn't contain any blood vessels—which carry blood cells, oxygen, and nutrients—it can't heal on its own. But with the right surgery, we can help fix articular cartilage damage. (medical practice) |
| 10 | What companies are involved in cartilage tissue engineering? (fact, education) | Top Cartilage repair Companies 1. Cytoseek. Private Company. Founded 2017. ... 2. Histogen Starts. Listed Company. Founded 2007. ... 3. NANOCHON. Private Company. Founded 2013. ... 4. Turn Biotechnologies. Private Company. ... 5. Carmell Therapeutics. Private Company. ... 6. Excellims Corporation. Private Company. ... 7. Lazzaro Medical. Private Company. ... 8. Xintela. Private Company. (Social Media) |
| 11 | What are the four common biomaterials? (fact, education) | Examples of biomaterials include metals, ceramics, glass, and polymers. (academic) |
| 12 | What is the scaffold for cartilage tissue  engineering? (fact, education) | Natural Polymers as Scaffolds for Cartilage Tissue Engineering. Natural  polymers, such as chitosan, collagen, alginate, silk fibroin, hyaluronan,  and gelatin, have been used extensively in TE for cartilage regeneration. (academic) |
| 13 | How do you reconstruct cartilage? (fact, technical details) | The procedure can be done with an arthroscope. A sharp tool called an awl is used to make multiple holes in the exposed bone surface, called subchondral bone. This action creates a healing response. New blood supply is able to reach the joint surface, bringing with it new cells that will form the new cartilage. (medical practice) |
| 14 | How do they regrow cartilage? (fact, technical details) | MACI is a surgical procedure that uses cartilage-forming cells from your body to restore damaged cartilage in the knees. It involves a biopsy to harvest chondrocytes (cartilage-forming cells), which are allowed to multiply in a lab, and surgery to implant them into the damaged area.(medical practice) |
| 15 | What is the difference between regenerative  engineering and tissue engineering? (fact, education) | Regenerative medicine is a broad field that includes tissue engineering but also incorporates research on self-healing – where the body uses its own systems, sometimes with help foreign biological material to recreate cells and rebuild tissues and organs. (government) |
| 16 | What are the benefits of cartilage regeneration? (fact, education) | The benefits of cartilage restoration include: Avoiding the need of placing artificial substances within the joint to provide pain relief. Possibly preventing the development of arthritis due to bone and joint injury. Helping people return to active lifestyles. (single doctor practice) |
| 17 | Why is tissue engineering the future? (value, evaluation of treatment) | The field of tissue engineering is a promising one, emerged as a technique to promote, direct and induce the innate capacity of tissues for regeneration and to assist in regaining function and shape, where the chances of natural healing are not possible. (government) |
| 18 | What are the advantages of collagen tissue  engineering? (fact, education) | Collagen can be extracted and purified from a variety of sources and offers low immunogenicity, a porous structure, good permeability, biocompatibility and biodegradability. (government) |
| 19 | What are two advantages of tissue engineering? (fact, education) | In medicine, tissue engineering can be used especially for the treatment of large bone or skin defects and can be considered as a valuable alternative for the re-implantation of bone or skin tissue as retrieved from other body sites. (government) |
| 20 | How successful is cartilage replacement? (value, evaluation of treatment) | Success rate: 95 percent after five years, 71 percent after 10 years, 66 percent after 20 years. (medical practice) * Surgical success rates generally reflect improvement in pain and activity levels. |
