# Supplementary material for: AI dialogues in cartilage repair: which guides evidence-based decisions better?
Source: Front Cell Dev Biol. 2026 May 29;14:1768270. doi: 10.3389/fcell.2026.1768270 (PMC13260151; doi:10.3389/fcell.2026.1768270)
Supplement: Supplementary file 3 [file Table8.docx]

| **Supplementary Table 3.** Answers to top 10 questions of Google Web Search from Deepseek | | |
| --- | --- | --- |
|  | question | Answer |
| 1 | What is the tissue engineering approach for cartilage? (fact, education) | Cartilage tissue engineering is a biomedical strategy aimed at repairing or regenerating damaged cartilage, which has limited self-healing capacity. The approach typically integrates three key components: scaffolds, cells, and growth factors.  1. Scaffolds: These provide a three-dimensional structure for cell attachment and proliferation. Recent advances focus on bionic scaffolds that mimic the natural extracellular matrix of cartilage, using materials like hydrogels, polymers, and decellularized matrices to create a supportive microenvironment.  2. Cells: Stem cells are commonly used as seed cells. For instance, autologous mesenchymal stem cells (MSCs) are isolated from the patient, expanded in culture, and seeded onto scaffolds. They are then induced to differentiate into chondrocytes using specific factors.  3. Growth Factors: Bioactive molecules such as TGF-β, BMPs, and IGF are delivered to regulate cell behavior and promote the synthesis of cartilage-specific matrix components like collagen type II and aggrecan.  Key Challenge: The main hurdle is producing engineered cartilage with mechanical properties and long-term stability comparable to native articular cartilage. Ongoing research focuses on optimizing scaffold design, cell sources, and signaling protocols to achieve functional integration. (Academic) |
| 2 | What are the challenges of cartilage tissue engineering? (fact, restriction) | Cartilage tissue engineering faces several significant hurdles. First, **avascularity** is a major challenge; cartilage lacks blood vessels, which severely limits nutrient and oxygen diffusion into engineered constructs, leading to core degradation and necrosis. Second, replicating the **zonal architecture** of native cartilage—with its distinct superficial, middle, and deep layers—is extremely difficult, as engineered scaffolds often fail to mimic this complex structural gradient.  Third, achieving **functional mechanical properties** is problematic. Engineered cartilage often lacks the compressive strength and low friction coefficient of native tissue, making it prone to failure under physiological loads. Fourth, **chondrocyte dedifferentiation** during in vitro expansion poses a risk; when chondrocytes are grown in monolayer, they lose their phenotype and produce inferior extracellular matrix. Finally, **integration with host tissue** remains poor, as engineered constructs often fail to bond seamlessly with surrounding native cartilage, leading to implant loosening and failure.  (academic) |
| 3 | Why is cartilage tissue engineering important? (fact, education) | Cartilage tissue engineering is critically important because **cartilage has minimal intrinsic healing capacity** due to its avascular nature—once damaged, it rarely regenerates, leading to chronic pain and joint degeneration. This has major clinical implications: over 30% of adults over 65 suffer from osteoarthritis, a condition often driven by progressive cartilage loss for which current treatments offer only symptomatic relief or temporary repair.  Second, current clinical options—such as microfracture, osteochondral autografts, and prosthetic joint replacement—are limited. Microfracture often produces inferior fibrocartilage, grafts risk donor site morbidity, and joint replacements have finite lifespans, especially problematic for younger, active patients. Tissue engineering aims to generate **bioresorbable, functional cartilage** that can integrate seamlessly with native tissue, potentially delaying or avoiding the need for artificial joint replacements.  Third, beyond orthopedics, cartilage engineering could address **tracheal, nasal, and auricular defects**, where autologous cartilage is scarce and carving synthetic implants is imprecise. If successful, this technology would reduce the dependence on donor tissue and artificial prostheses, lower revision surgery rates, and improve patients' long-term quality of life by restoring native-like joint function. (academic) |
| 4 | How cells biomaterial or tissue engineering can be used for cartilage regeneration? (policy, indications) | Cartilage regeneration through tissue engineering integrates three core components: cells, biomaterials (scaffolds), and bioactive signals, often called the "triad" of tissue engineering.  **Cells** are the primary building blocks. Two main cell types are used: autologous chondrocytes (harvested from the patient's own cartilage) and mesenchymal stem cells (MSCs) derived from bone marrow, adipose tissue, or synovium. MSCs are particularly promising because they can differentiate into chondrocytes under appropriate biochemical stimuli, such as TGF-β growth factor supplementation.  **Biomaterials** serve as temporary scaffolds that mimic the native extracellular matrix. Natural polymers like collagen, hyaluronic acid, and alginate are frequently used due to their biocompatibility, while synthetic polymers such as poly (lactic-co-glycolic acid) (PLGA) offer tunable degradation rates and mechanical strength. Advanced scaffolds now incorporate **porosity and mechanical gradients** to match native cartilage's zonal architecture, and some are designed as injectable hydrogels for minimally invasive delivery.  **Tissue engineering** combines cells and scaffolds with bioreactor culture or in vivo signals. For instance, chondrocytes or MSCs can be seeded onto a collagen scaffold and cultured in a bioreactor that applies cyclic compression, mechanically stimulating the cells to produce cartilage-specific extracellular matrix rich in collagen type II and aggrecan. Clinically, this approach has been applied in matrix-induced autologous chondrocyte implantation (MACI), where a cell-seeded scaffold is implanted into the cartilage defect.  (government) |
| 5 | What biomaterials are used in cartilage tissue engineering? (fact, education) | Biomaterials used in cartilage tissue engineering fall into three main categories: natural polymers, synthetic polymers, and composite/hybrid scaffolds.  **Natural polymers** are widely used due to their excellent biocompatibility and bioactivity. Common examples include **collagen** (the main protein in native cartilage), **hyaluronic acid** (a key glycosaminoglycan), **alginate** (derived from seaweed), **chitosan** (from crustacean shells), and **agarose**. These materials naturally mimic the extracellular matrix of cartilage and promote cell adhesion and differentiation, though they often lack sufficient mechanical strength.  **Synthetic polymers** offer tunable mechanical properties and controlled degradation rates. Key examples include **poly(lactic-co-glycolic acid) (PLGA)**, **polycaprolactone (PCL)**, and **poly(ethylene glycol) (PEG)** hydrogels. These can be engineered to match the compressive modulus of native cartilage and degrade over weeks to months as new tissue forms. However, they may lack the bioactive cues that natural polymers provide.  **Composite/hybrid scaffolds** combine natural and synthetic materials to overcome individual limitations. For instance, PLGA reinforced with collagen or hyaluronic acid can achieve both mechanical strength and bioactivity. Additionally, **hydrogels**—especially injectable hydrogels—are gaining popularity because they can be delivered minimally invasively and fill irregular defect shapes, with materials like hyaluronic acid hydrogels and PEG hydrogels being prominent examples. (academic) |
| 6 | What is the technology for cartilage replacement? (fact, technical details) | Cartilage replacement technology has advanced significantly in recent years, offering several approaches to repair damaged articular cartilage. One prominent method is autologous chondrocyte implantation (ACI), where a patient's own cartilage cells are harvested, cultured, and re-implanted into the defect. More recent innovations include stem cell-based implants like Plurocart, which uses stem cells to regenerate cartilage tissue. Another cutting-edge approach is the use of acellular allografts that provide a structural scaffold for the body's own cells to repopulate and integrate, as demonstrated in integrative cartilage repair research. In 2024, the FDA approved a new generation of knee cartilage repair product, marking a significant milestone in the field. Furthermore, products like CARTIHEAL AGILI-C have shown superior 5-year results in improving knee pain and function by promoting natural cartilage regeneration. These technologies move beyond traditional joint replacement by aiming to restore the native cartilage structure and function, potentially delaying or preventing the need for total joint arthroplasty. (government) |
| 7 | Why can't we repair cartilage? (fact, restrictions) | Cartilage has a notoriously limited capacity for self-repair due to several intrinsic biological factors. First and foremost, articular cartilage is **avascular**—it lacks blood vessels. Without a direct blood supply, the tissue cannot deliver the necessary nutrients, oxygen, and progenitor cells to the injury site, and it fails to mount a standard inflammatory healing response. Additionally, cartilage is **aneural and alymphatic**, meaning it lacks nerves and lymphatic drainage, further limiting its repair signaling.  Another key obstacle is that **chondrocytes** (the resident cartilage cells) have a very low mitotic activity and are trapped within a dense extracellular matrix, preventing them from migrating to the site of injury. Furthermore, the mature cartilage matrix is dense and impermeable, which physically blocks cell movement. The tissue also lacks a robust population of accessible stem or progenitor cells capable of generating new cartilage.  Finally, even when some repair is attempted, the resulting tissue is often **fibrocartilage** rather than the smooth, durable hyaline cartilage, which is biomechanically inferior and prone to degeneration over time. These combined factors—avascularity, low cellular activity, matrix entrapment, and inadequate progenitor cells—explain why cartilage cannot heal itself effectively once damaged. (academic) |
| 8 | What is the most difficult tissue to engineer? (fact, education) | Among the various tissues targeted for regenerative medicine, **articular cartilage** is widely considered one of the most difficult to engineer successfully. Despite decades of research, cartilage regeneration remains elusive primarily because of its unique biological and mechanical characteristics. Unlike bone, which has a robust intrinsic healing capacity, cartilage is **avascular, aneural, and alymphatic**. This lack of blood supply severely limits the delivery of nutrients, oxygen, and reparative cells to the injury site, and prevents the tissue from mounting a standard inflammatory healing response.  Furthermore, the **chondrocytes** (cartilage cells) are sparse, have low metabolic activity, and are encased within a dense extracellular matrix, making them unable to migrate to damaged areas. Even when laboratory-grown constructs are implanted, they often fail to replicate the zonal architecture and biomechanical properties of native hyaline cartilage—such as its compressive stiffness, low friction, and durability under repetitive load-bearing. The resulting tissue frequently degrades into weaker fibrocartilage over time.  Other tissues, such as **cardiac muscle** and **nervous tissue**, are also notoriously difficult to engineer due to their poor regenerative capacity and complex functional requirements. However, cartilage's combination of avascularity, low cellularity, high mechanical demands, and vulnerability to degeneration makes it arguably the most challenging target in tissue engineering. (government) |
| 9 | Why is cartilage so hard to repair? (fact, education) | Cartilage is exceptionally difficult to repair due to a combination of biological and structural limitations that distinguish it from most other tissues in the body.  The primary reason is **avascularity**—cartilage lacks blood vessels entirely. Without a direct blood supply, the tissue cannot deliver the necessary immune cells, nutrients, and progenitor cells to the injury site needed to mount a standard healing response. This absence of vascularity also prevents the formation of a blood clot, which is the first step in most tissue repair processes.  Second, **chondrocytes** (the resident cartilage cells) are sparse and have an extremely low metabolic and mitotic rate. These cells are also trapped within a dense extracellular matrix, limiting their ability to migrate to the site of injury. Even when chondrocytes attempt repair, they often produce **fibrocartilage** rather than the original hyaline cartilage, which is mechanically inferior and degenerates over time.  Third, cartilage is **aneural**, meaning it lacks nerves, so early damage often goes unnoticed, delaying treatment. Additionally, the tissue has a **limited number of endogenous progenitor cells** that could be activated for repair. Finally, because cartilage is subjected to high mechanical loads and repetitive stress, any repaired tissue must withstand these forces immediately, a requirement that most engineered or naturally repaired tissue cannot meet.  These factors—avascularity, low cellular activity, matrix entrapment, poor progenitor supply, and high mechanical demands—collectively explain why cartilage remains one of the most challenging tissues in the body to repair successfully. (government) |
| 10 | What companies are involved in cartilage tissue engineering? (fact, education) | Several notable companies are actively involved in cartilage tissue engineering. Japan Tissue Engineering Co., Ltd. (J-TEC) is a pioneer in regenerative medicine, developing cultured autologous cartilage products for clinical applications  1. Hyalex Orthopaedics focuses on creating synthetic, biomimetic implants that mimic the mechanical properties of healthy cartilage for joint repair  2. Axcelda specializes in 3D bioprinting technologies to produce cell-based therapies specifically designed to treat cartilage injuries  3. Sparta Biomedical is developing a bioengineered cartilage implant intended to regenerate articular cartilage and restore joint function  4. Altach is working on innovative solutions for cartilage repair, leveraging advanced biomaterials and tissue engineering approaches 5.  These companies represent a diverse range of strategies, including autologous cell transplantation, synthetic biomaterial implants, and 3D bioprinting of living tissues, all aimed at addressing the significant clinical challenge of cartilage regeneration. The accuracy of this information may change over time as companies update their pipelines and products. (Medical Practice) |
