# Supplementary material for: AI dialogues in cartilage repair: which guides evidence-based decisions better?
Source: Front Cell Dev Biol. 2026 May 29;14:1768270. doi: 10.3389/fcell.2026.1768270 (PMC13260151; doi:10.3389/fcell.2026.1768270)
Supplement: Supplementary file 5 [file Table7.docx]

| **Supplementary Table 6.** Answers to top 10 questions of Google Web Search from ChatGPT | | |
| --- | --- | --- |
|  | question | Answer |
| 1 | What is the best surgery for cartilage repair?  (Policy, Indications/Management) | According to a systematic review of cartilage repair techniques, **there is no single “best” surgery for cartilage repair**, because outcomes depend on defect size, patient age, and activity level. However, commonly used procedures include microfracture, autologous chondrocyte implantation (ACI), and osteochondral autograft transplantation.  For small cartilage defects, **microfracture surgery** is often recommended due to its simplicity and short-term effectiveness. It stimulates bone marrow to form repair tissue, but this tissue is typically fibrocartilage and may deteriorate after 18–24 months.  For larger defects (>4 cm²) or in younger, active patients, **autologous chondrocyte implantation (ACI)** is often considered a better option. Evidence suggests ACI may provide more durable, long-term outcomes compared to microfracture, although it requires a two-stage procedure.  **Osteochondral autograft transplantation (OAT)** can offer rapid improvement and good long-term results for localized defects, but it is limited by donor-site morbidity.  Overall, current evidence indicates that all techniques can improve symptoms, but **ACI may be preferable for larger lesions, while microfracture remains suitable for smaller ones**. Importantly, high-quality studies have not definitively identified a universally superior procedure. (government) |
| 2 | What is the surgical repair of cartilage? (Fact, Education) | According to an educational review on cartilage surgery, **the surgical repair of cartilage refers to a group of operative techniques designed to restore damaged articular cartilage and re-establish a functional joint surface**.  These procedures are generally classified into three categories: **palliation, repair, and restoration**. Palliative techniques (e.g., chondroplasty) aim to relieve symptoms without fully restoring cartilage. Repair techniques, such as fixation of cartilage fragments or microfracture, attempt to stimulate the body’s natural healing response. For example, microfracture creates small holes in the subchondral bone to release bone marrow cells that form new, though often inferior, fibrocartilage.  Restorative procedures aim to regenerate or replace cartilage more effectively. These include **autologous chondrocyte implantation (ACI)**, where a patient’s cartilage cells are cultured and reimplanted, and **osteochondral grafting (autograft or allograft)**, where healthy cartilage and bone are transplanted into the defect.  The overall goal of surgical cartilage repair is to **restore joint congruity, reduce pain, and prevent long-term degeneration such as osteoarthritis**. However, no single technique is universally superior, and the choice of procedure depends on factors such as defect size, location, and patient characteristics. (academic) |
| 3 | How to repair cartilage surgery? (Fact, Technical details) | According to a review in the Journal of Orthopaedic Surgery and Research, cartilage repair surgery involves a series of clinical steps aimed at restoring damaged articular cartilage depending on defect size and patient factors.  First, the surgeon evaluates the lesion through imaging (e.g., MRI) and arthroscopy to determine its size, depth, and location. Small defects are often treated with microfracture, where tiny holes are created in the subchondral bone to stimulate marrow-derived stem cells and form repair tissue.  For moderate lesions, osteochondral autograft transplantation (OAT) may be used, transferring healthy cartilage and bone from a non-weight-bearing area to the defect site. Larger or more complex defects may require autologous chondrocyte implantation (ACI), in which cartilage cells are harvested, expanded in vitro, and reimplanted into the defect.  After the procedure, rehabilitation is critical, involving controlled weight-bearing and physical therapy to promote integration and functional recovery. Outcomes vary depending on technique and patient characteristics, and no single method is universally superior. (academic) |
| 4 | What is the success rate of cartilage repair? (Value, Evaluation) | According to a systematic review of cartilage repair outcomes, the success rate of cartilage repair varies by surgical technique, patient factors, and follow-up duration, and no single procedure guarantees consistent results across all cases.  For autologous chondrocyte implantation (ACI), long-term data show approximately 82% of patients achieve successful outcomes, although about 18% may experience failure or require revision surgery.  For microfracture, short-term results are often favorable, with clinical improvement in many patients, but durability may decline over time due to the formation of fibrocartilage rather than native hyaline cartilage.  Other techniques, such as osteochondral autograft transplantation (OAT) and matrix-assisted ACI (MACI), also demonstrate relatively high success rates, often reported in the range of 70–90% clinical improvement or patient satisfaction, although results vary depending on lesion size and patient selection.  Importantly, outcomes are influenced by factors such as age, defect size, joint alignment, and rehabilitation adherence. Younger patients with smaller, isolated lesions tend to have better results, while larger or degenerative lesions are associated with lower success rates.  Overall, current evidence suggests that cartilage repair procedures are generally effective in improving symptoms, but long-term success remains variable and technique-dependent, and high-quality comparative studies are still limited. (academic) |
| 5 | What age can you repair cartilage? (Policy, Indications/Management) | According to NCBI, cartilage repair can be performed across a wide age range, but patient age significantly influences outcomes rather than eligibility itself.  In general, younger patients (typically under 40–50 years old) tend to achieve better clinical results because they have greater regenerative capacity, healthier subchondral bone, and fewer degenerative changes. Procedures such as autologous chondrocyte implantation (ACI) and osteochondral autograft transplantation (OAT) are often preferred in this group, especially for focal cartilage defects.  In contrast, older patients, particularly those with osteoarthritis, may have less favorable outcomes due to diffuse cartilage degeneration and reduced healing potential. In these cases, cartilage repair techniques may be less effective, and alternative treatments such as joint replacement may be considered. However, selected older individuals with isolated defects and good joint condition may still benefit from repair procedures.  Overall, there is no strict age cutoff, but most evidence suggests that cartilage repair is most successful in younger, active patients with localized lesions, while outcomes become more variable with increasing age. (government) |
| 6 | Can you fix cartilage without surgery? (Policy, Indications/Management) | According to a review in Osteoarthritis and Cartilage, cartilage has a very limited intrinsic healing capacity, and it generally cannot fully repair itself without surgical intervention, especially in articular joints.  However, non-surgical (conservative) treatments can help manage symptoms and support joint function. These include physical therapy, weight management, activity modification, and medications such as nonsteroidal anti-inflammatory drugs (NSAIDs). In some cases, injections (e.g., corticosteroids or hyaluronic acid) may reduce pain and improve mobility, although they do not restore cartilage structure.  Emerging approaches such as biologic therapies (e.g., platelet-rich plasma or stem cell-based injections) have shown potential for symptom relief, but current evidence remains limited and inconsistent regarding true cartilage regeneration.  Overall, while non-surgical treatments may alleviate symptoms and slow progression, they typically do not achieve complete structural repair of cartilage, and surgical options may be required for significant defects. (academic) |
| 7 | Can diet affect cartilage repair? (Value, Evaluation) | According to current biomedical research, diet can **indirectly influence cartilage repair**, but it does not directly “regrow” cartilage on its own.  Cartilage is avascular (lacks blood vessels), so its ability to self-repair is very limited. However, nutritional factors can shape the metabolic and inflammatory environment that affects chondrocytes (cartilage cells). Diets rich in **anti-inflammatory nutrients** (e.g., omega-3 fatty acids, antioxidants, and adequate protein) may help reduce joint inflammation and slow cartilage degeneration, while excessive saturated fat or obesity-related metabolic changes can worsen cartilage damage through inflammatory pathways.  Scientific reviews also highlight that lipid metabolism, influenced by diet, plays a role in cartilage homeostasis—proper lipid balance may support cartilage function, while dysregulated metabolism promotes osteoarthritis progression.  However, no clinical evidence shows that diet alone can regenerate lost cartilage in humans. At best, diet can **support the biological conditions for repair therapies** (such as exercise, physical therapy, or regenerative medicine) and potentially slow further deterioration.  In summary, diet affects cartilage repair indirectly by modulating inflammation and metabolic health, but it is not a standalone method for restoring damaged cartilage. (academic) |
| 8 | Is cartilage surgery painful? (Value, Pain) | According to clinical orthopedic and rehabilitation literature, cartilage surgery is not painless, but the pain is usually expected, temporary, and medically manageable.  Because most cartilage procedures (such as arthroscopy, microfracture, or meniscus repair) are minimally invasive, the operation itself is performed under anesthesia, so patients do not feel pain during surgery. However, after the procedure, post-operative pain, swelling, and stiffness are common, especially in the first few days when inflammation peaks.  Pain intensity depends on the specific procedure. For example, cartilage repair or meniscus surgery can cause moderate soreness and tightness, often requiring pain medication, ice, elevation, and physiotherapy. In most cases, pain gradually improves over 1–6 weeks, though full recovery may take months depending on the repair type.  Importantly, studies show arthroscopic procedures are generally less painful than open joint surgery, with faster recovery and fewer complications.  In summary, cartilage surgery does involve post-operative pain, but it is typically short-term, controlled with medication, and decreases steadily during recovery rather than being severe long-term pain.  (medical practice) |
| 9 | Can I walk after cartilage surgery? (Fact, Time of recovery) | According to orthopedic rehabilitation guidelines, whether you can walk after cartilage surgery depends strongly on the type and location of the procedure.  For minimally invasive procedures such as knee arthroscopy or small cartilage debridement, patients are often allowed to walk on the same day or within 24 hours, usually with crutches for support. However, weight-bearing may be limited to protect the healing cartilage.  For more complex cartilage restoration procedures—such as microfracture, autologous chondrocyte implantation (ACI), or osteochondral grafting—walking is typically restricted for several weeks. Patients may need to avoid full weight-bearing for 2–6 weeks or longer to allow cartilage repair tissue to mature properly.  Physical therapy is usually started early, focusing first on range-of-motion exercises before progressing to partial and then full weight-bearing.  In summary, you may be able to walk shortly after minor cartilage surgery, but significant cartilage repair procedures often require delayed or partial walking to protect healing tissue. (medical practice) |
| 10 | How long does cartilage repair surgery take? (Fact, Time of recovery) | According to clinical orthopedic surgery references, the duration of cartilage repair surgery typically ranges from about 30 minutes to 2–3 hours, depending on the specific technique and joint condition.  For simple arthroscopic cartilage procedures (such as debridement or minor smoothing), the operation may take 30–60 minutes. These are minimally invasive and usually performed as outpatient procedures.  For more advanced cartilage restoration techniques—such as microfracture, autologous chondrocyte implantation (ACI), or osteochondral grafting—surgery can take 1.5 to 3 hours, since they involve more complex preparation, implantation, or graft fixation steps.  If multiple procedures are combined (e.g., ligament repair plus cartilage reconstruction), the operative time may extend further.  In summary, cartilage repair surgery is generally not very long in absolute surgical time, but complexity varies widely: simple arthroscopy is short (under 1 hour), while biological cartilage reconstruction procedures may take several hours. (academic) |
