# Supplementary material for: AI dialogues in cartilage repair: which guides evidence-based decisions better?
Source: Front Cell Dev Biol. 2026 May 29;14:1768270. doi: 10.3389/fcell.2026.1768270 (PMC13260151; doi:10.3389/fcell.2026.1768270)
Supplement: Supplementary file 6 [file Table2.docx]

| **Supplementary Table 2.** Top 20 Q&As from ChatGPT | | |
| --- | --- | --- |
|  | question | Answer |
| 1 | What is cartilage tissue engineering? (fact,education) | According to the National Institute of Arthritis and Musculoskeletal and Skin Diseases (NIAMS), cartilage tissue engineering is a multidisciplinary field that focuses on creating functional cartilage tissue through a combination of biology, engineering, and materials science. The primary goal of cartilage tissue engineering is to develop innovative strategies to repair, regenerate, or replace damaged or degenerated cartilage in the body. This field involves the design and implementation of various techniques and materials to stimulate the growth of new cartilage tissue in a controlled and organized manner. (government) |
| 2 | How does cartilage tissue engineering work? (fact, technical details) | According to the National Institute of Biomedical Imaging and Bioengineering (NIBIB), cartilage tissue engineering is a field of research that aims to create functional cartilage tissue to repair or replace damaged or degenerated cartilage in the body. The process typically involves the following steps: 1. Cell Isolation: Cells are usually isolated from the patient's own body, such as chondrocytes (cartilage cells) or mesenchymal stem cells (MSCs). These cells serve as the building blocks for the new cartilage tissue. 2. Cell Expansion: The isolated cells are then cultured and expanded in a laboratory setting to increase their numbers. This step is crucial to obtain a sufficient quantity of cells for tissue engineering. 3. Scaffold Selection: A scaffold material is chosen to provide structural support for the cells and guide their growth into the desired cartilage shape. Scaffolds can be made from a variety of biocompatible materials, such as synthetic polymers or natural materials like collagen. 4. Cell Seeding: The expanded cells are then seeded onto the scaffold, where they attach and begin to proliferate. This forms the initial structure for the new cartilage tissue. 5. Bioreactor Culture: The scaffold with the seeded cells is placed in a bioreactor, which provides the necessary environmental conditions for cell growth and tissue maturation. This can include controlling factors like temperature, oxygen levels, and nutrient supply. Maturation and Integration: Over time, the seeded cells continue to grow and differentiate into mature cartilage tissue. Once the tissue reaches an appropriate level of maturity, it can be implanted into the patient's damaged area. 6. Monitoring and Follow-Up: After implantation, patients are monitored to ensure proper integration and function of the engineered cartilage. Follow-up care may include physical therapy to optimize the healing process. Please note that the field of tissue engineering is continually evolving, and there may have been advancements and refinements in the techniques and approaches since my last update in September 2021.（government) |
| 3 | What are the applications of cartilage tissue engineering? (policy, indication) | Articular Cartilage Repair: One of the primary applications is the repair of damaged or degenerated articular cartilage, which covers the ends of bones in joints. This is especially relevant for individuals with osteoarthritis or cartilage injuries due to trauma. 1. Meniscus Regeneration: Tissue engineering techniques can be used to regenerate the meniscus, a wedge-shaped cartilage structure in the knee joint. Meniscal injuries are common, and engineered meniscal tissue can help restore joint function. 2. Nasal and Ear Reconstruction: Cartilage tissue engineering has been employed in reconstructive surgeries for the nose and ears. Engineered cartilage can provide the necessary structural support and shape in cases of congenital defects or traumatic injuries. 3. Tracheal and Laryngeal Reconstruction: Tissue engineering techniques are being explored for the reconstruction of cartilaginous structures in the respiratory system, such as the trachea and larynx, to address conditions like tracheal stenosis or cancer-related defects. 4. Craniofacial and Jaw Reconstruction: Cartilage tissue engineering has applications in craniofacial surgery for the reconstruction of facial features, especially in cases of craniofacial anomalies or after tumor removal. 5. Auricular (Ear) Cartilage Reconstruction: Synthetic or engineered cartilage can be used to create or reconstruct external ear structures, making it valuable in plastic and reconstructive surgery. 6. Research and Drug Testing: Engineered cartilage tissue models are used for drug testing and basic research, allowing scientists to study cartilage physiology, disease mechanisms, and potential treatments. 7. Biomechanical Testing: Engineered cartilage constructs are valuable for biomechanical studies, helping to better understand the mechanical properties of cartilage and the performance of tissue-engineered replacements. 8. Tissue Banking: Engineered cartilage can be preserved in tissue banks for potential future use in transplantation procedures. (government) |
| 4 | Can cartilage tissue engineering be used to treat osteoarthritis? (policy, indication) | Cartilage tissue engineering is a promising field that holds the potential to address cartilage-related conditions, including osteoarthritis. Osteoarthritis is a degenerative joint disease characterized by the progressive breakdown of articular cartilage. While there is ongoing research in this area, it's important to note that as of my last knowledge update in September 2021, cartilage tissue engineering for osteoarthritis is still an area of active investigation and development. According to the Arthritis Foundation, cartilage tissue engineering approaches are being explored as a means to treat osteoarthritis. The idea is to regenerate or replace damaged cartilage with engineered tissue, potentially providing a more effective and longer-lasting solution compared to traditional treatments such as pain management or joint replacement surgery. （Social Media） |
| 5 | What materials are used in cartilage tissue engineering? (fact, technical details) | According to a review article, a variety of materials are used in cartilage tissue engineering. These materials serve as scaffolds to support cell growth and tissue formation, and they can be broadly categorized into three main types: 1. Natural Polymers: ·Collagen: Collagen is the most abundant protein in native cartilage and is commonly used as a scaffold material due to its biocompatibility and ability to mimic the native extracellular matrix (ECM). ·Chitosan: Chitosan is derived from chitin and offers biocompatibility and biodegradability. It can be modified to improve its mechanical properties. ·Hyaluronic Acid (HA): HA is a major component of the synovial fluid in joints. It is often used to enhance the hydrophilicity and lubrication properties of scaffold materials. 2. Synthetic Polymers: ·Poly(lactic-co-glycolic acid) (PLGA): PLGA is a synthetic polymer with tunable mechanical properties and degradation rates. It is commonly used in tissue engineering due to its versatility. ·Poly(ethylene glycol) (PEG): PEG is a hydrophilic polymer that can be functionalized with various bioactive molecules. It is used to enhance scaffold biocompatibility. ·Poly(ε-caprolactone) (PCL): PCL is known for its mechanical strength and slow degradation, making it suitable for long-term applications. 3. Hybrid Materials: ·Some scaffolds combine both natural and synthetic materials to leverage the advantages of each. For instance, a blend of collagen and PLGA can offer improved mechanical properties and bioactivity. （academic) |
| 6 | Are there different approaches to cartilage tissue engineering? (fact, technical details) | According to the National Institute of Arthritis and Musculoskeletal and Skin Diseases (NIAMS), there are indeed various approaches to cartilage tissue engineering. These approaches are designed to address the complex challenges associated with regenerating or repairing cartilage, especially in the context of cartilage injuries or degenerative conditions like osteoarthritis. Some of the key approaches include: 1. Cell-Based Approaches: These methods involve using specific cell types, such as chondrocytes (cartilage cells) or mesenchymal stem cells (MSCs), to generate new cartilage tissue. 2. Scaffold-Based Approaches: Scaffolds, made from natural or synthetic materials, serve as structural support for cell growth and tissue formation.  3. Biomimetic Materials: Biomimetic materials are engineered to closely mimic the composition and properties of native cartilage tissue.  4. Growth Factors and Biologics: Growth factors and bioactive molecules are used to stimulate cell differentiation and encourage the production of the extracellular matrix (ECM) necessary for cartilage structure.  5. Bioreactors and Mechanical Stimulation: Bioreactors are specialized systems that provide a controlled environment for the maturation of engineered cartilage tissue.  6. Hybrid Approaches: Many tissue engineering strategies combine elements from multiple approaches to optimize outcomes, such as using both cells and scaffolds. (government) |
| 7 | What are the challenges in cartilage tissue engineering? (fact, restriction) | According to the National Center for Biotechnology Information (NCBI), cartilage tissue engineering faces several significant challenges that researchers and clinicians are actively working to address. Some of the primary challenges in cartilage tissue engineering include: 1. Biomechanical Properties: Replicating the complex biomechanical properties of native cartilage, including its load-bearing capacity and resilience, remains a challenge.  2. Integration with Surrounding Tissues: Achieving seamless integration between the engineered cartilage and the surrounding native tissue is difficult.  3. Vascularization: Cartilage is an avascular tissue, meaning it lacks blood vessels. However, engineered tissues need a blood supply for nutrients and oxygen.  4. Long-Term Viability: Sustaining the viability and functionality of engineered cartilage over extended periods is a challenge. Degradation and loss of mechanical properties can occur over time. 5. Quality and Consistency: Ensuring consistent quality and reproducibility in cartilage tissue engineering remains an ongoing concern, as there can be variability in the properties of engineered tissue constructs. 6. Regulatory Hurdles: The development and clinical translation of tissue-engineered cartilage face regulatory challenges, which include demonstrating safety, efficacy, and quality control to meet regulatory standards for medical products. 7. Patient-Specific Approaches: Tailoring treatments to individual patient needs is a growing area of interest but poses challenges in terms of cost-effectiveness, scalability, and personalized medicine. 8. Ethical and Legal Issues: Ethical considerations regarding the use of cells, biologics, and intellectual property rights can impact the development and commercialization of cartilage tissue engineering products. (academic) |
| 8 | Is cartilage tissue engineering a viable alternative to traditional joint  replacement surgery? (policy, indication) | Cartilage tissue engineering holds promise as an alternative to traditional joint replacement surgery in certain cases, especially for individuals with localized cartilage defects or early-stage osteoarthritis. Joint replacement surgery typically involves removing the damaged joint and replacing it with an artificial prosthesis. While joint replacement is highly successful in providing pain relief and improved joint function, it has limitations, such as a finite lifespan for the prosthetic joint and potential complications associated with surgery. According to the National Institute of Arthritis and Musculoskeletal and Skin Diseases (NIAMS), cartilage tissue engineering aims to repair and regenerate damaged cartilage, potentially preserving the natural joint and avoiding the need for a full joint replacement.  However, it's important to note that cartilage tissue engineering is not a one-size-fits-all solution. Its effectiveness can depend on factors like the size and location of the cartilage defect, the patient's age, and overall joint health. Additionally, the field is still evolving, and further research is needed to optimize outcomes and expand its applicability. Ultimately, the choice between traditional joint replacement surgery and cartilage tissue engineering should be made on a case-by-case basis in consultation with a healthcare provider. (government) |
| 9 | What are the advantages of using tissue-engineered cartilage? (fact, education) | Tissue-engineered cartilage offers several advantages: 1. Natural Tissue Preservation: Unlike traditional joint replacement surgery, tissue engineering aims to preserve natural joint tissue, reducing the need for complete joint replacement. This can help maintain joint function and integrity. 2. Customization: Tissue engineering allows for the creation of personalized cartilage grafts tailored to the patient's specific needs, including size, shape, and location of defects. 3. Less Invasive: The implantation of tissue-engineered cartilage is typically a less invasive procedure than joint replacement, leading to reduced postoperative pain, shorter recovery times, and potentially fewer complications. 4. Longevity: When successful, tissue-engineered cartilage can have a longer lifespan compared to traditional artificial joint replacements, which may wear out over time. 5. Biocompatibility: Engineered cartilage scaffolds are designed to be biocompatible, minimizing the risk of rejection or adverse reactions. 6. Youthful Candidates: Tissue engineering is particularly beneficial for younger patients with localized cartilage defects, as it offers a regenerative solution that can delay or eliminate the need for joint replacement later in life. 7. Improved Quality of Life: Successful tissue-engineered cartilage can lead to improved joint function, reduced pain, and enhanced overall quality of life for patients. While tissue-engineered cartilage holds promise, it's essential to note that it is not a universal solution and its success depends on various factors, including patient-specific conditions and the extent of cartilage damage. (academic) |
| 10 | Are there any successful clinical cases of cartilage tissue engineering? (value, Evaluation of treatment) | According to the literature and scientific research available up to my knowledge cutoff date in September 2021, there have been successful clinical cases of cartilage tissue engineering. Cartilage tissue engineering is a promising field that aims to regenerate or repair damaged cartilage using various techniques and biomaterials. One notable example is the use of autologous chondrocyte implantation (ACI). ACI is a technique where a patient's own chondrocytes (cartilage cells) are harvested, expanded in vitro, and then re-implanted into the damaged area. This approach has shown success in treating cartilage defects in the knee, hip, and other joints. Clinical trials and long-term follow-up studies have demonstrated improved patient outcomes and cartilage regeneration. Additionally, matrix-assisted autologous chondrocyte implantation (MACI) is an advancement in this field. It involves the use of a cell-seeded scaffold to support the regeneration of cartilage. Clinical trials have shown promising results with MACI in the treatment of cartilage defects. While these techniques have shown success in clinical cases, it's important to note that the field of cartilage tissue engineering continues to evolve with ongoing research, and newer approaches and technologies may have emerged since my last update in September 2021. It's advisable to consult more recent scientific literature and consult with medical professionals for the latest developments in this field. (academic) |
| 11 | How long does it take to grow cartilage tissue using tissue  engineering techniques? (fact, timeline of recovery) | According to existing research and medical literature up to my last knowledge update in September 2021, the time it takes to grow cartilage tissue using tissue engineering techniques can vary depending on several factors. These factors include the specific techniques used, the extent of cartilage damage, and the type of tissue engineering approach employed. Here are some general considerations: The time required to grow cartilage tissue using tissue engineering techniques varies, typically ranging from several weeks to several months. It depends on factors such as the chosen technique (e.g., autologous chondrocyte implantation, stem cell-based approaches), the size and location of the defect, and the desired tissue maturity. For instance, autologous chondrocyte implantation involves several weeks for cell expansion and cultivation, followed by additional months for tissue integration. Advances in research may eventually lead to more efficient methods with shorter timelines, but currently, cartilage tissue engineering remains a process that requires careful planning and patience to achieve successful results. (academic) |
| 12 | What are the potential risks or complications of cartilage tissue  engineering? (policy, risks) | Potential risks and complications of cartilage tissue engineering include: 1. Incomplete Regeneration: The engineered tissue may not fully replicate the structural and functional properties of natural cartilage. 2. Infection: Surgical procedures for implantation can introduce the risk of infection. 3. Immune Response: The body may react to implanted cells or materials, leading to inflammation or rejection. 4. Graft Failure: The implanted tissue may not integrate well with the surrounding tissue or may degrade over time. 5. Hypertrophy: Overgrowth of the engineered tissue can occur, altering joint mechanics. 6. Scar Tissue Formation: In some cases, fibrous tissue may form instead of cartilage. 7. Long-Term Durability: The longevity of engineered cartilage may be uncertain, and revision surgeries may be needed. Patients and healthcare providers must carefully assess the benefits and risks before pursuing cartilage tissue engineering procedures. (government) |
| 13 | Can cartilage tissue engineering be used for other joints besides  the knee? (fact, education) | Yes, cartilage tissue engineering can be used for other joints besides the knee. While knee joints are a common focus due to the prevalence of knee injuries, tissue engineering techniques have been explored for joints such as the hip, shoulder, ankle, and even smaller joints like those in the hand and wrist. The principles of cartilage regeneration remain applicable, with adjustments made for the specific anatomy and mechanical demands of each joint. However, the success and feasibility of cartilage tissue engineering in these joints can vary, and research and clinical applications continue to expand in this area. (government) |
| 14 | Are there ethical considerations in cartilage tissue engineering research? (policy, risks) | Yes, according to National Institutes of Health (NIH), there are ethical considerations in cartilage tissue engineering research. Key concerns include the sourcing of cells, particularly stem cells, with proper informed consent and without coercion. There are also ethical considerations related to animal testing, as well as the potential commercialization of regenerative therapies, which could lead to unequal access to treatments. Ensuring patient safety, transparency in research, and adherence to ethical guidelines is crucial in this field. Additionally, balancing the promise of cartilage tissue engineering with potential long-term effects and patient expectations is an ongoing ethical challenge for researchers and healthcare professionals. (government) |
| 15 | What are the current advancements in cartilage tissue engineering? (fact, education) | according to scientific literature and medical research, advancements in cartilage tissue engineering were focused on improving the quality and durability of engineered cartilage tissue. Some key areas of progress included: 1. 3D Bioprinting: The use of 3D bioprinting technology has advanced, allowing for precise placement of cells and materials to create complex cartilage structures. 2. Stem Cell Therapies: Improved methods for differentiating stem cells into chondrocytes have been developed, enhancing the quality of engineered cartilage. 3. Biomaterial Innovations: Researchers are exploring novel biomaterials that mimic the native cartilage environment more closely. 4. Mechanical Stimulation: Incorporating mechanical cues during tissue culture enhances the development of functional cartilage. 5. Patient-Specific Solutions: Advancements in personalized medicine enable the creation of patient-specific cartilage grafts. These advancements hold promise for improving the success, durability, and accessibility of cartilage tissue engineering, potentially leading to better treatments for joint injuries and degeneration. However, I recommend checking the latest scientific literature for the most up-to-date developments in the field. (academic) |
| 16 | Is cartilage tissue engineering available as a mainstream medical treatment? (value, evaluation of treatment) | cartilage tissue engineering had not yet become a mainstream medical treatment, but it was a field showing promising progress. Clinical trials and research efforts were underway to develop effective cartilage tissue engineering therapies. The adoption of these therapies would depend on several factors, including regulatory approvals, long-term safety and efficacy data, and widespread acceptance within the medical community. To get the most current information on the status of cartilage tissue engineering as a mainstream medical treatment, I recommend checking recent medical journals, clinical guidelines, and consulting with healthcare professionals or experts in the field. Advances in medical treatments can occur rapidly, and the status of cartilage tissue engineering may have evolved since my last update in 2021. (academic) |
| 17 | Are there any FDA-approved cartilage tissue engineering products? (fact, accessibility) | As of my last update in September 2021, the FDA (U.S. Food and Drug Administration) had approved certain cartilage tissue engineering products for clinical use. One notable example is MACI (Matrix-Induced Autologous Chondrocyte Implantation), which involves the use of a patient's own cultured chondrocytes on a membrane scaffold to repair cartilage defects in the knee. Additionally, other autologous chondrocyte implantation (ACI) products and approaches have received FDA approval or clearance for specific applications. However, the availability of FDA-approved cartilage tissue engineering products may have evolved since then, so it's advisable to check the latest updates and consult with healthcare professionals for the most current information. (government) |
| 18 | How is cartilage tissue engineering different from other tissue engineering approaches? (fact, education) | Based on the information from the field of tissue engineering and the unique characteristics of cartilage, cartilage tissue engineering differs from other tissue engineering approaches in several key ways: 1. Tissue-Specific Challenges: Cartilage presents specific challenges not found in other tissues, such as avascularity (lack of blood vessels), limited self-healing capacity, and susceptibility to mechanical wear and tear. These challenges necessitate specialized approaches for cartilage regeneration. 2. Cell Types: Cartilage tissue engineering predominantly relies on chondrocytes (cartilage-specific cells) or stem cells with chondrogenic potential. 3. Biomaterials and Scaffolds: Biomaterials and scaffolds used in cartilage tissue engineering are designed to mimic the unique extracellular matrix of cartilage. These materials differ from those used in other tissue engineering projects, which adapt to the specific needs of the targeted tissue. 4. Biomechanical Considerations: Cartilage experiences distinct mechanical forces compared to other tissues. Therefore, cartilage tissue engineering often employs specialized bioreactors to replicate these forces and support tissue maturation. 5. Growth Factors: Cartilage tissue engineering utilizes growth factors tailored to chondrogenesis, differing from the growth factors employed in the regeneration of other tissue types. 6. Clinical Applications: Cartilage tissue engineering is primarily applied to treat cartilage defects resulting from conditions like osteoarthritis or injuries. In contrast, other tissue engineering approaches have diverse applications, ranging from wound healing to organ transplantation. 7. Immunomodulation: Managing the immune response is critical in cartilage tissue engineering to prevent graft rejection. Strategies for immunomodulation may vary between cartilage regeneration and other tissue engineering projects. 8. Long-Term Stability: Achieving long-term stability and functional performance of engineered cartilage is a unique challenge due to its need to withstand mechanical loads without vascular support.. (academic) |
| 19 | What are the prospects for personalized cartilage tissue engineering? (fact, education) | According to the National Institute of Biomedical Imaging and Bioengineering (NIBIB), the prospects for personalized cartilage tissue engineering are highly promising. Personalization allows treatments to be tailored to an individual's specific cartilage defects, improving effectiveness and long-term outcomes. Innovations in 3D printing, biomaterials, and patient-specific stem cells offer the means to create customized implants that match the patient's anatomy precisely. However, challenges related to cost-effectiveness, regulatory approvals, and long-term efficacy monitoring must be addressed. As research and technology continue to evolve, personalized cartilage tissue engineering is expected to play an increasingly pivotal role in enhancing the treatment of cartilage injuries and degenerative conditions. (government) |
| 20 | Can stem cells be used in cartilage tissue engineering? (policy, indication) | Yes, stem cells are a valuable component in cartilage tissue engineering. Mesenchymal stem cells (MSCs), derived from sources like bone marrow, adipose tissue, or umbilical cord, can differentiate into chondrocytes, the cells responsible for cartilage formation. These stem cells are often used in combination with scaffolds and growth factors to promote cartilage regeneration. Stem cell-based approaches have shown promise in repairing cartilage defects, improving tissue quality, and offering a potential source for autologous cell therapy. However, challenges such as ensuring proper differentiation, maintaining cell viability, and achieving consistent outcomes still need to be addressed for widespread clinical use. (academic) |
