# Supplementary material for: AI dialogues in cartilage repair: which guides evidence-based decisions better?
Source: Front Cell Dev Biol. 2026 May 29;14:1768270. doi: 10.3389/fcell.2026.1768270 (PMC13260151; doi:10.3389/fcell.2026.1768270)
Supplement: Supplementary file 7 [file Table5.docx]

| **Supplementary Table 5.** Top 20 Q&As from Google Web Search | | |
| --- | --- | --- |
|  | question | Answer |
| 1 | What is the best surgery for cartilage repair?  (Policy, Indications/Management) | The OATS procedure, also called mosaicplasty, involves taking healthy cartilage from non-weight-bearing areas of the joint and transplanting it into the damaged areas. Because the transfer happens within the same joint, this procedure works best for small areas of damaged cartilage. (academic) |
| 2 | What is the surgical repair of cartilage? (Fact, Education) | What is cartilage repair? Cartilage repair includes various surgical procedures such as microfracture, osteochondral autograft transfer (also known as OATS or Mosaicplasty), osteochondral allograft transplantation and autologous chondrocyte implantation. (commercial) |
| 3 | How to repair cartilage surgery? (Fact, Technical details) | Microfracture. The surgeon drills small holes in the bone and bone marrow under the damaged articular cartilage. These holes stimulate the bone to repair itself, triggering cells from the bone marrow to repair the damaged cartilage. The newly formed cartilage tissue, however, is fibrocartilage. (medical practice) |
| 4 | What is the success rate of cartilage repair? (Value, Evaluation) | Knee cartilage restoration techniques have come a long way since they were first introduced. Today, patients can expect success rates up to 80% to 90% for some procedures. (medical practice) |
| 5 | What age can you repair cartilage? (Policy, Indications/Management) | Significant improvements in outcomes are consistently seen for patients aged >40 years among all cartilage restoration procedures, including MFx, ACI, and OAT. However, most authors with experience and expertise in the field of cartilage repair recommend an age limit between 40 and 50 years. (commercial) |
| 6 | Can you fix cartilage without surgery? (Policy, Indications/Management) | NYU Langone doctors often recommend nonsurgical options as a first-line treatment for a knee cartilage injury. These include anti-inflammatory medication, physical therapy, and injections into the joint, including steroids; hyaluronic acid, also known as synthetic joint fluid; and platelet-rich plasma (PRP). (academic) |
| 7 | Can diet affect cartilage repair? (Value, Evaluation) | Additionally, because collagen is a protein, the protein content of legumes helps replenish the collagen necessary to rebuild cartilage. And, as if all of that weren't enough, legumes also have high levels of lysine, an amino acid that is critical to cartilage regeneration. (medical practice) |
| 8 | Is cartilage surgery painful? (Value, Pain) | Knee cartilage repair surgery is done under general anaesthetic, which means you'll be asleep for the full operation and won't feel anything, and typically lasts around an hour. (medical practice) |
| 9 | Can I walk after cartilage surgery?  (Fact, Time of recovery) | You may not be able to put weight on your leg for up to a week after your surgery, so you may need some help to stand or walk. Your hospital may give you crutches or a knee brace to take home. Your surgeon or nurse will give you advice on how to use these, and for how long. (medical practice) |
| 10 | How long does cartilage repair surgery take? (Fact, Time of recovery) | Simpler arthroscopic surgeries such as chondroplasty and microfracture taken about 45 to 60 minutes. Cartilage restoration procedures such as OATS, OCA and MACI are open surgeries and therefore typically take roughly 2 hours. (government) |
| 11 | What are the risks of cartilage surgery? (Policy, Risk/Complication) | Knee cartilage restoration is a relatively safe procedure; however, as with any surgery, there are risks and complications that could occur, such as: Post-operative bleeding. Blood clots (deep vein thrombosis).  (medical practice) |
| 12 | What exercises should I avoid with cartilage damage?  (Policy, Indications/Management) | - Squats. Squatting exercises, particularly deep squats, cause excessive strain on the knees, making them a poor choice for those with knee pain. ... - Lunges. ... - Leg Extensions. ... - High-Intensity Interval Training (HIIT) ... - Jumping Exercises. ... - Stair Climbing. ... (medical practice) |
| 13 | Why is cartilage not repaired? (Fact, Education) | Cartilage in adults doesn't naturally repair itself by generating new cells that form new tissue. Your cartilage isn't vascularized, meaning no blood vessels carry proteins and other nutrients into the soft tissue. (social media) |
| 14 | Does glucosamine rebuild cartilage? (Value, Evaluation) | Glucosamine sulphate and glucosamine hydrochloride are nutritional supplements. Animal studies have found that glucosamine can both delay the breakdown of and repair damaged cartilage. The results for the use of glucosamine for osteoarthritis are mixed and the size of the effect is modest. (government) |
| 15 | How is cartilage surgery done? (Fact, Technical details) | The procedure can be done with an arthroscope. Multiple holes are made through the injured area in the subchondral bone with a surgical drill or wire. The subchondral bone is penetrated to create a healing response. Like microfracture, drilling yields fibrocartilage, not hyaline cartilage. (single doctor practice) |
| 16 | What is the best treatment for cartilage repair? (Policy, Indications/Management) | Sometimes, physiotherapy may be recommended to help reduce pain and improve movement and strength in the knee. If the damage is severe, you may need arthroscopy surgery to repair or remove the damaged cartilage. (academic) |
| 17 | What is the best exercise for knee cartilage damage? (Policy, Indications/Management) | **Exercises for knee problems**   - Lying knee bend. ... - Static quad strengthening. ... - Supported leg raise. ... - Lying leg raise. ... - Seated knee extension stretch.   (academic) |
| 18 | How to repair cartilage without surgery? (Value, Evaluation of treatment) | **Non-Surgical options for knee cartilage restoration**   1. Physical Therapy and Exercise. ... 2. Platelet-Rich Plasma (PRP) Injections. ... 3. Hyaluronic Acid (HA) Injections. ... 4. Stem Cell Therapy. ... 5. Dietary Supplements. ... 6. Lifestyle Modifications.   (government) |
| 19 | What to expect after cartilage surgery? (Fact, Cost) | Limited weight bearing activities, full knee extension by 2-3 weeks. Independent home exercise, including stationary bike and balancing exercises, as early as one month. Full weight bearing and full knee range of motion by 8-12 weeks post-surgery. Free from knee brace by 8-12 weeks post-surgery. (medical practice) |
| 20 | How to prepare for cartilage surgery? (Policy, Indications/Management) | 3 Days before surgery. Confirm the exact location and time of surgery. Arrange for transportation as you will not be able to drive home.  1 Day before surgery. Follow your orthopedic surgeon's recommendations regarding food, drink, and medications.  (commercial) |
