# Supplementary material for: AI dialogues in cartilage repair: which guides evidence-based decisions better?
Source: Front Cell Dev Biol. 2026 May 29;14:1768270. doi: 10.3389/fcell.2026.1768270 (PMC13260151; doi:10.3389/fcell.2026.1768270)
Supplement: Supplementary file 9 [file Table3.docx]

| **Supplementary Table 3.** Answers to top 10 questions of Google Web Search from ChatGPT | | |
| --- | --- | --- |
|  | question | Answer |
| 1 | What is the tissue engineering approach for cartilage? (fact, education) | According to the National Institute of Arthritis and Musculoskeletal and Skin Diseases (NIAMS), the approach for cartilage regeneration typically involves several key steps: 1. Cell Selection: Begin by selecting appropriate cells for cartilage repair. Chondrocytes, the specialized cells present in cartilage, are preferred. Mesenchymal stem cells (MSCs) are also commonly used, as they can be induced to differentiate into chondrocytes. 2. Biomaterials and Scaffolds: Utilize biomaterials and scaffolds that mimic the extracellular matrix (ECM) of cartilage. These structures provide mechanical support and help guide tissue growth. Materials like hydrogels, nanocomposites, or 3D-printed constructs are often employed. 3. Biomechanical Stimulation: Subject the engineered cartilage to controlled mechanical loading using specialized bioreactors. This mimics the forces natural cartilage experiences, encouraging tissue maturation and strength. 4. Growth Factors: Implement growth factors like transforming growth factor-beta (TGF-β) or bone morphogenetic proteins (BMPs) to promote chondrogenesis and the synthesis of cartilage-specific extracellular matrix components. 5. Implantation: Once the engineered cartilage is matured, it can be implanted into the patient's damaged area, such as an osteochondral defect or articular cartilage injury. 6. Immunomodulation: Manage the immune response to the implanted tissue, partic*ul*arly when allogeneic grafts are used, to prevent graft rejection. (government) |
| 2 | What are the challenges of cartilage tissue engineering? (fact, restriction) | According to a review article by Makris, Gomoll, Malizos, Hu, and Athanasiou in 2015, the challenges of cartilage tissue engineering encompass several aspects: 1. Cell Source and Proliferation: Choosing an optimal cell source that is capable of extensive proliferation and maintaining the chondrogenic phenotype during expansion is difficult. 2. Scaffold Design: Developing a scaffold that can support chondrocyte function, provide appropriate mechanical properties, and degrade at a rate that matches tissue formation is complex. 3. Mechanical Properties: Achieving the mechanical properties of native cartilage, which are crucial for joint function, remains a substantial challenge. 4. Vascularization: Since cartilage is an avascular tissue, engineering a tissue that can survive and function without blood vessels is difficult. 5. Integration: Ensuring seamless integration between the newly formed tissue and the surrounding native cartilage to restore joint functionality is critical and challenging. 6. In Vivo Environment: Mimicking the in vivo environment, including biochemical, mechanical, and spatial cues, to guide cell behavior in vitro is challenging. 7. Scale-Up: Scaling up the laboratory-scale processes to produce larger tissue constructs without compromising the quality and functionality of the engineered cartilage is a notable challenge. 8. Regulatory Compliance: Meeting regulatory requirements for safety, efficacy, and quality in translating the tissue-engineered products from bench to bedside is a complex process. (academic) |
| 3 | Why is cartilage tissue engineering important? (fact, education) | According to a review article titled "Cartilage tissue engineering: From biomaterials and stem cells to osteoarthritis treatments", cartilage tissue engineering has diverse applications in regenerative medicine and orthopedics. It's used to address various conditions, including osteoarthritis, joint injuries, and cartilage defects in the knee, hip, and shoulder. It's also employed in reconstructive surgeries for nasal and ear deformities, meniscus tears, and tracheal and laryngeal issues.  Researchers explore its potential for spinal disc regeneration, craniofacial reconstruction, and even cosmetic enhancements. Cartilage tissue engineering offers promising solutions to improve joint function, reduce pain, and restore cartilage in the body. Its applications continue to expand as advancements in science and technology open new avenues for regenerative treatments, benefiting individuals with cartilage-related conditions and injuries. (academic) |
| 4 | How cells biomaterial or tissue engineering can be used for cartilage regeneration? (policy, indications) | Yes, cartilage tissue engineering holds great promise as a potential treatment for osteoarthritis (OA). OA is a degenerative joint condition characterized by the gradual breakdown of cartilage, leading to pain and reduced joint mobility. Cartilage tissue engineering aims to address this by replacing or repairing damaged cartilage. Researchers are exploring various approaches, such as using stem cells (e.g., mesenchymal stem cells) to generate new cartilage tissue in the laboratory. These engineered cartilage tissues can then be transplanted into the affected joints. This process has shown potential in preclinical and clinical studies for improving joint function and reducing OA-related pain. Additionally, cartilage tissue engineering techniques are being used to develop scaffolds and biomaterials that can stimulate. (government) |
| 5 | What biomaterials are used in cartilage tissue engineering? (fact, education) | 1. Biodegradable Polymers: Polymers like poly(lactic-co-glycolic acid) (PLGA) and polyethylene glycol (PEG) are commonly used as scaffolds to support cartilage growth. They provide mechanical support and gradually degrade as the new tissue forms. 2. Hydrogels: Hydrogels, such as agarose and alginate, mimic the natural environment of cartilage due to their high water content. They can encapsulate cells and support their growth while offering good biocompatibility. 3. Extracellular Matrix (ECM) Components: Natural cartilage ECM components like collagen and chondroitin sulfate are often incorporated into scaffolds to enhance cell attachment and tissue regeneration. 4. Cells: Chondrocytes (cartilage cells) or stem cells, such as mesenchymal stem cells (MSCs), are seeded onto the scaffolds to promote cartilage formation. 5. Growth Factors: Growth factors like TGF-β and BMPs are used to stimulate cell proliferation and differentiation. (academic) |
| 6 | What is the technology for cartilage replacement? (fact, technical details) | Yes, there are various approaches to cartilage tissue engineering. According to the National Center for Biotechnology information (NCBI), The three primary strategies include: 1. Cell-Based Approach: This method involves seeding chondrocytes or stem cells onto a scaffold, providing them with a supportive environment for cartilage formation. 2. Scaffold-Free Approach: In this approach, cells are cultured without a scaffold, relying on cell-to-cell interactions to produce cartilage tissue. 3. Biomaterial-Based Approach: Biomaterials like hydrogels and biodegradable polymers are used as scaffolds to facilitate cartilage regeneration. These scaffolds can be combined with cells and growth factors to enhance tissue formation. The choice of approach depends on factors such as the extent of cartilage damage, the desired outcome, and the available resources for tissue engineering applications. (government) |
| 7 | Why can't we repair cartilage? (fact, restrictions) | According to an article from the National Center for Biotechnology Information (NCBI), articular cartilage has a limited ability to repair and self-renew due to its avascular nature, meaning it lacks blood vessels, and its low cell density. The cartilage experiences constant mechanical stresses, making it vulnerable to wear, tears, and injuries. Current treatment options, while able to improve joint function and reduce pain, do not effectively regenerate the articular tissue with its distinct functional characteristics. Therefore, strategies for repair are only partially effective and often result in the formation of mechanically inferior cartilage. Advanced regenerative technologies are being explored to inform future treatment options and enhance cartilage repair and regeneration. (government) |
| 8 | What is the most difficult tissue to engineer? (fact, education) | According to an article from the National Center for Biotechnology Information (NCBI), tissue engineering can be utilized to restore, maintain, or enhance tissues and organs. However, the potential impact of this field is broad and complex. The article discusses the exploitation of living cells in various ways to achieve these goals, with visions of building organs from scratch in the laboratory for transplantation into patients. The article also touches upon the challenges and opportunities in tissue engineering, such as the technical and biological complexities of replicating the intricate structures and functions of native tissues. The engineering of certain tissues, like the heart or organs with complex structures and functions, can be particularly challenging due to the need to replicate not only the cellular structure but also the organ's functionality, vascularization, and integration with the host's body. (government) |
| 9 | Why is cartilage so hard to repair? (fact, education) | Tissue-engineered cartilage offers several advantages: 1. Natural Tissue Preservation: Unlike traditional joint replacement surgery, tissue engineering aims to preserve natural joint tissue, reducing the need for complete joint replacement. This can help maintain joint function and integrity. 2. Customization: Tissue engineering allows for the creation of personalized cartilage grafts tailored to the patient's specific needs, including size, shape, and location of defects. 3. Less Invasive: The implantation of tissue-engineered cartilage is typically a less invasive procedure than joint replacement, leading to reduced postoperative pain, shorter recovery times, and potentially fewer complications. 4. Longevity: When successful, tissue-engineered cartilage can have a longer lifespan compared to traditional artificial joint replacements, which may wear out over time. 5. Biocompatibility: Engineered cartilage scaffolds are designed to be biocompatible, minimizing the risk of rejection or adverse reactions. 6. Youthful Candidates: Tissue engineering is particularly beneficial for younger patients with localized cartilage defects, as it offers a regenerative solution that can delay or eliminate the need for joint replacement later in life. Improved Quality of Life: Successful tissue-engineered cartilage can lead to improved joint function, reduced pain, and enhanced overall quality of life for patients. While tissue-engineered cartilage holds promise, it's essential to note that it is not a universal solution and its success depends on various factors, including patient-specific conditions and the extent of cartilage damage. (academic) |
| 10 | What companies are involved in cartilage tissue engineering? (fact, education) | Several companies are involved in cartilage tissue engineering, exploring innovative approaches to repair and regenerate cartilage tissue. Here are a few of them: 1. Histogenics: Histogenics is a regenerative medicine company that combines cell therapy and tissue engineering technologies to develop innovative products for tissue repair. 2. NANOCHON: NANOCHON is another company listed under cartilage repair, though specific details about their approaches in cartilage tissue engineering might be found on their official website or related publications. 3. EpiBone: EpiBone is focused on engineering bone, cartilage, and osteochondral tissues from stem cells, working to translate innovations toward clinical use. Dr. Bhumiratana, one of the team members, holds patents and has authored books related to the field. These companies utilize various strategies, including the use of biomaterials, stem cells, and innovative technologies, to develop solutions for cartilage repair and regeneration. For detailed information about their research, technologies, and products, you might explore their respective websites or scientific publications. (social media) |
