# Supplementary material for: AI dialogues in cartilage repair: which guides evidence-based decisions better?
Source: Front Cell Dev Biol. 2026 May 29;14:1768270. doi: 10.3389/fcell.2026.1768270 (PMC13260151; doi:10.3389/fcell.2026.1768270)
Supplement: Supplementary file 10 [file Table4.docx]

| **Supplementary Table 4.** Top 20 Q&As from DeepSeek | | |
| --- | --- | --- |
|  | question | Answer |
| 1 | What is cartilage repair surgery?  (Fact, Education) | A surgical procedure to repair, replace, or regenerate damaged articular cartilage in a joint (most commonly the knee, but also hip, ankle, and shoulder) to relieve pain and restore function. (academic). |
| 2 | Who is a good candidate for cartilage repair surgery?  (Policy, Indications/Management) | Typically, younger patients (often under 50) with a single, focal cartilage defect, stable ligaments, good joint alignment, and who have not found relief from non-surgical treatments (physical therapy, injections, etc.). (academic) |
| 3 | What are the different types of cartilage repair surgery?  (Fact, Technical details) | Common types include Microfracture, OATS (Osteochondral Autograft Transfer System), ACI (Autologous Chondrocyte Implantation), MACI (Matrix-Induced Autologous Chondrocyte Implantation), Osteochondral Allograft Transplantation, and DeNovo NT Graft. |
| 4 | How long is the recovery time for cartilage repair surgery?  (Fact, Timeline of recovery) | Recovery is lengthy and varies by procedure. It often involves weeks of non-weight bearing with crutches, months of physical therapy, and a full return to high-impact sports can take 6 to 12 months or longer.  (medical practice) |
| 5 | What is the success rate of cartilage repair surgery?  (Value, Evaluation of treatment) | Success rates are generally high (often 80-90% for reducing pain and improving function), but they depend heavily on the patient's age, the size/location of the defect, the specific procedure used, and adherence to rehabilitation. (academic) |
| 6 | Is cartilage repair surgery painful?  (Value, Pain) | As with any major surgery, there is a significant post-operative pain period that is managed with medications. The extensive rehabilitation process can also be challenging and uncomfortable. (medical practice) |
| 7 | What is the cost of cartilage repair surgery?  (Fact, Cost) | Cost varies widely by procedure, location, and insurance. It can range from $10,000 to $50,000+. Insurance often covers medically necessary procedures after conservative treatments fail. (academic) |
| 8 | What is microfracture surgery? (Fact, Technical details) | A common technique where small holes are drilled into the bone beneath the cartilage defect to release marrow cells, which form a "super clot" that becomes fibrocartilage (a scar-tissue-like substitute for true cartilage). (academic) |
| 9 | What is the difference between microfracture and MACI?  (Fact, Technical details) | Microfracture stimulates the body to create fibrocartilage. MACI is a two-step procedure: first, healthy cartilage cells are harvested; then, they are grown in a lab and implanted onto a scaffold to regenerate more durable, hyaline-like cartilage. (Academic) |
| 10 | Can cartilage grow back or regenerate after surgery?  (Fact, Education) | Articular cartilage has a very poor natural ability to heal. The goal of surgery is to stimulate the growth of new tissue (either fibrocartilage or, ideally, hyaline-like cartilage) to fill the defect.  (Academic) |
| 11 | What happens if you don't repair cartilage damage?  (Policy, Indications/Management) | Unrepaired cartilage defects often worsen over time. The damaged area can expand, leading to increased pain, swelling, joint instability, and significantly accelerating the progression of osteoarthritis. (Academic) |
| 12 | How is cartilage damage diagnosed?  (Fact, Technical details) | Through a physical exam, patient history, and imaging. An MRI is the gold standard non-invasive method for visualizing soft tissue like cartilage. Diagnosis is sometimes confirmed via arthroscopy. (Academic) |
| 13 | What are the risks and complications of cartilage surgery?  (Policy, Risks/complication) | Risks include infection, blood clots, nerve or blood vessel damage, joint stiffness, failure of the graft to incorporate, and the growth of abnormal repair tissue (arthrofibrosis). (Academic) |
| 14 | Will I need a knee replacement after cartilage surgery?  (Policy, Indications/Management) | The primary goal of cartilage repair is to delay or prevent the need for a joint replacement, especially in younger patients. It is a joint-preserving procedure. (Academic) |
| 15 | Can you walk after cartilage repair surgery?  (Fact, Restriction) | Not immediately. Patients are almost always required to be non-weight bearing on the affected leg for 4-8 weeks to protect the healing graft. Walking is gradually reintroduced with physical therapy guidance. (Academic) |
| 16 | What is an osteochondral allograft?  (Fact, Technical details) | A procedure where a donor graft of healthy bone and cartilage is transplanted into the prepared defect site. It's often used for larger defects. (Academic) |
| 17 | What is the role of physical therapy in recovery?  (Policy, Indications/Management) | Physical therapy is critical. It begins immediately to reduce swelling and restore range of motion and progresses over many months to strengthen the muscles around the joint, protect the repair, and gradually restore function. (Academic) |
| 18 | Are there any new innovative treatments for cartilage repair? (Value, Evaluation of treatment) | Yes, the field is rapidly evolving. New techniques include BioCartilage (minced cartilage fragments), stem cell injections (often combined with other procedures), and AMIC (Autologous Matrix-Induced Chondrogenesis), which combines microfracture with a collagen scaffold. (Academic) |
| 19 | Can cartilage surgery be performed arthroscopically?  (Fact, Technical details) | Some procedures, like microfracture or debridement, are routinely done arthroscopically (minimally invasive). More complex procedures like OATS, ACI, or allograft transplantation often require an open incision (arthrotomy). (Commercial) |
| 20 | What are the alternatives to surgery for cartilage damage? (Policy, Indications/Management) | Alternatives include PRP (Platelet-Rich Plasma) injections, cortisone injections, hyaluronic acid (gel) injections, physical therapy, activity modification, and unloader braces. These are typically tried first unless the defect is severe. (Commercial) |
