# Supplementary material for: AI dialogues in cartilage repair: which guides evidence-based decisions better?
Source: Front Cell Dev Biol. 2026 May 29;14:1768270. doi: 10.3389/fcell.2026.1768270 (PMC13260151; doi:10.3389/fcell.2026.1768270)
Supplement: Supplementary file 11 [file Table6.docx]

| **Supplementary Table 6.** Answers to top 10 questions of Google Web Search from DeepSeek R1 | | |
| --- | --- | --- |
|  | question | Answer |
| 1 | What is the best surgery for cartilage repair?  (Policy, Indications/Management) | There is no single "best" surgery for cartilage repair, as the ideal choice depends heavily on the defect's size, location, and the patient's age. For smaller defects (<2 cm²), microfracture or autologous matrix-induced chondrogenesis (AMIC) are common. For larger, more challenging defects, autologous chondrocyte implantation (ACI) or osteochondral autograft transplantation (OATS/Mosaicplasty) are preferred. ACI uses lab-grown patient cells to regenerate tissue, while OATS transplants healthy cartilage from a non-weight-bearing area. Ultimately, an orthopedic surgeon determines the optimal procedure based on a comprehensive patient evaluation to ensure the best possible outcome. (government) |
| 2 | What is the surgical repair of cartilage? (Fact, Education) | The surgical repair of cartilage involves procedures to restore damaged articular cartilage—the smooth tissue cushioning joint surfaces. Common techniques include microfracture, where small holes are drilled into bone to stimulate blood flow and new cartilage growth; autologous chondrocyte implantation (ACI), which transplants lab-cultured patient-owned cartilage cells into defects; and osteochondral grafting, transferring healthy cartilage and bone plugs from non-weight-bearing areas to injured sites. These methods aim to reduce pain, improve joint function, and delay or prevent arthritis progression, often benefiting younger patients with localized injuries rather than widespread joint degeneration. (academic) |
| 3 | How to repair cartilage surgery? (Fact, Technical details) | Cartilage repair surgery involves techniques to regenerate or replace damaged joint cartilage. Common procedures include microfracture, where surgeons create tiny fractures in the underlying bone to release marrow cells that form new cartilage-like tissue. Autologous chondrocyte implantation (ACI) involves harvesting healthy cartilage cells from the patient, culturing them in a lab, and implanting them into the damaged area. Osteochondral grafting transplants healthy cartilage and bone from a non-weight-bearing joint region to the injured site. These minimally invasive, often arthroscopic surgeries aim to relieve pain, restore function, and delay joint replacement. Recovery requires strict rehabilitation to protect the healing tissue. (academic) |
| 4 | What is the success rate of cartilage repair? (Value, Evaluation) | The success rate of cartilage repair surgery varies significantly based on the specific procedure, the size and location of the defect, the patient's age, and activity level. Generally, **short-term success rates** (2-5 years) for techniques like microfracture or autologous chondrocyte implantation (ACI) range from **70% to 85%** in terms of pain reduction and functional improvement. However, long-term outcomes can decline, with some studies showing durability concerns beyond 10 years, particularly for larger defects. Success is higher in younger patients with small, traumatic defects in stable joints. Factors like obesity, misalignment, or advanced arthritis reduce efficacy, often necessitating further intervention, including joint replacement. (academic) |
| 5 | What age can you repair cartilage? (Policy, Indications/Management) | Cartilage repair is typically most effective for younger patients (teens to adults in their 40s or 50s) with focal cartilage defects due to injury rather than widespread arthritis. While there is no strict upper age limit, success depends on biological factors like healing capacity and joint health. Older patients with advanced osteoarthritis or degenerative changes are often less ideal candidates, as their cartilage may lack regenerative potential, and joint replacement may offer more reliable outcomes. Eligibility is determined case-by-case based on activity level, defect size, joint stability, and overall bone quality, not chronological age alone. (medical practice) |
| 6 | Can you fix cartilage without surgery? (Policy, Indications/Management) | Yes, non-surgical options can help manage cartilage damage, though they cannot fully "fix" or regenerate severely damaged cartilage. Conservative treatments include physical therapy to strengthen muscles around the joint, reducing pressure on cartilage; pain management with anti-inflammatory medications (e.g., NSAIDs); and injections like corticosteroids for temporary pain relief or hyaluronic acid to lubricate the joint. Emerging alternatives like platelet-rich plasma (PRP) or stem cell injections may promote healing in mild cases by harnessing the body’s natural repair mechanisms. However, these are often palliative and most effective for early-stage wear or partial tears, not advanced degeneration. (medical practice) |
| 7 | Can diet affect cartilage repair? (Value, Evaluation) | While diet cannot directly regenerate cartilage, it plays a supportive role in creating an optimal environment for repair and reducing inflammation. Key nutrients like protein (for collagen synthesis), vitamin C (collagen formation), omega-3 fatty acids (anti-inflammatory effects), and antioxidants (e.g., vitamins A and E) may aid tissue healing and slow cartilage degradation. Conversely, processed foods, sugars, and saturated fats can exacerbate inflammation, potentially impairing recovery. For surgical cartilage repair, a balanced diet supports overall health and healing but is not a standalone solution. It complements medical interventions rather than replacing them. (medical practice) |
| 8 | Is cartilage surgery painful? (Value, Pain) | Yes, cartilage surgery involves postoperative pain, though its intensity and duration depend on the procedure's invasiveness and individual pain tolerance. Arthroscopic surgeries (e.g., microfracture or chondroplasty) typically cause moderate pain, swelling, and stiffness for days to weeks, managed with oral medications. Open procedures like osteochondral grafting or autologous chondrocyte implantation (ACI) often involve more pain due to larger incisions and bone involvement, requiring stronger analgesics. Pain peaks within the first 48 hours and gradually subsides with rest, ice, and physical therapy. While discomfort is expected, modern pain management protocols—including nerve blocks and multimodal analgesia—significantly reduce suffering during recovery. (medical practice) |
| 9 | Can I walk after cartilage surgery? (Fact, Time of recovery) | Yes, you can walk after cartilage surgery, but weight-bearing restrictions and the use of crutches are typically required to protect the healing tissue. For arthroscopic procedures like microfracture or chondroplasty, patients often use crutches for 2-6 weeks, with partial weight-bearing based on the surgeon’s protocol. More extensive repairs (e.g., osteochondral grafting or ACI) may require non-weight-bearing for up to 6-8 weeks to allow integration of grafts or cells. Early movement is encouraged to prevent stiffness, but strict adherence to guidelines is crucial to avoid graft failure or delayed healing. Rehabilitation progresses gradually from assisted walking to full weight-bearing over months. (medical practice) |
| 10 | How long does cartilage repair surgery take? (Fact, Time of recovery) | The duration of cartilage repair surgery varies based on the technique and complexity of the defect. Arthroscopic procedures like microfracture or chondroplasty typically take 30–60 minutes. More involved techniques, such as autologous chondrocyte implantation (ACI) or osteochondral grafting, require 1–2 hours due to the meticulous harvesting and transplantation of tissue. Open surgeries may extend longer if combined with alignment corrections (e.g., osteotomy). Factors like defect size, location, and patient-specific anatomy also influence timing. While operative time is relatively short, overall time in the operating room includes anesthesia setup and preoperative preparations. (medical practice) |
